# Long-term supplementation with 3200 to 4000 IU of vitamin D daily and adverse events: a systematic review and meta-analysis of randomized controlled trials

**DOI:** 10.1007/s00394-023-03124-w

**Published:** 2023-02-28

**Authors:** Armin Zittermann, Christian Trummer, Verena Theiler-Schwetz, Stefan Pilz

**Affiliations:** 1 Clinic for Thoracic and Cardiovascular Surgery, Herz- und Diabeteszentrum NRW, Ruhr University Bochum, Georgstraße 11, 32545 Bad Oeynhausen, Germany; 2 Department of Internal Medicine, Division of Endocrinology and Diabetology, Medical University of Graz, Graz, Austria

**Keywords:** Vitamin D, Hypercalcemia, Intoxication, Harmful effects, Upper tolerable intake level, Adverse events

## Abstract

**Purpose:**

The upper tolerable intake level for vitamin D in the general population has been set at 4000 international units (IU) daily, but considerable uncertainty remains. We summarized reported harmful effects of a daily vitamin D supplement of 3200–4000 IU in trials lasting ≥ 6 months.

**Methods:**

We performed a systematic review and meta-analysis of randomized controlled trials in several databases and identified 22 trials reporting safety data. Parameters of calcium metabolism, falls, hospitalization, and mortality were assessed.

**Results:**

The selected trials comprised a total number of 12,952 participants. All trials used supplemental vitamin D_3_. The relative risk (RR) of hypercalcemia in the vitamin D vs. control arm was 2.21 (95%CI: 1.26–3.87; 10 studies), with a vitamin D-induced frequency of hypercalcemia of 4 cases per 1000 individuals. Subgroup analysis in trials with > 100 and ≤ 100 study participants revealed an RR of 2.63 (95%CI: 1.30–5.30; 7 studies) and 0.80 (95%CI: 0.24–2.62; 3 studies), respectively (P_interaction_ = 0.06). Risks of falls and hospitalization were also significantly increased in the vitamin D arm with an RR of 1.25 (95%CI: 1.01–1.55; 4 studies) and 1.16 (95%CI: 1.01–1.33; 7 studies), respectively. Risks of hypercalciuria, kidney stones, and mortality did not differ significantly between study arms. Quality assessment revealed high risk of incomplete reporting of safety-related outcome data.

**Conclusion:**

Supplemental vitamin D doses of 3200–4000 IU/d appear to increase the risk of hypercalcemia and some other adverse events in a small proportion of individuals, indicating that this dose is not completely safe. In future studies, rigorous reporting of safety-related outcomes is needed when using moderately high doses of vitamin D.

**Supplementary Information:**

The online version contains supplementary material available at 10.1007/s00394-023-03124-w.

## Introduction

The concept of vitamin D safety consists of two models, the safe tolerable upper intake level (UL) method, and the idea of adequate, but not excessive, circulating 25-hydroxyvitamin D (25[OH]D) levels, the latter being the generally accepted indicator of vitamin D status [1]. For its 2011 report, the Institute of Medicine (IOM) has performed a rigorous literature search and has set the UL for vitamin D at 4000 international units (IU) daily for those aged 9 years or older [2]. This value was primarily based on case reports and assumed that hypercalcemia, the hallmark of vitamin D intoxication, is unlikely to occur at daily vitamin D doses below 10,000 IU. In addition, an uncertainty factor of 2.5 was applied for potential ethnic/racial differences in vitamin D susceptibility and other adverse clinical consequences that may occur at lower doses. In 2012, the European Food Safety Authority ESFA also set the UL for vitamin D at 4000 IU/d for those aged 11 years or older [3]. This UL is currently being re-evaluated [4].

With respect to circulating 25(OH)D, it was assumed that even after maximal sun exposure values generally remain below 125–150 nmol/L [2]. The IOM also took the observation into account that 6 months of daily vitamin D supplementation with 5000 IU resulted in circulating 25(OH)D concentrations between 100 and 150 nmol/l. In addition, because epidemiological data also indicated an inverse J-shaped association between 25(OH)D and morbidity and mortality risk, the IOM classified circulating 25(OH)D concentration greater than 125 nmol/l as potentially harmful. EFSA stated that studies reporting on an association between 25(OH)D concentration and all-cause mortality or cancer are inconsistent [3]. Likewise, the IOM stated that there was considerable uncertainty regarding the upper adequate 25(OH)D concentration [2].

Since 2011/2012, various randomized controlled trials (RCTs) have examined potentially beneficial effects of vitamin D on various organs and clinical outcomes. However, a recent systematic review and meta-analysis on long-term supplementation of large vitamin D doses also reported a trend for an increased risk of hypercalcemia [5]. Some other trials, in which high doses of vitamin D were administered intermittently, reported a higher risk of falls or fractures if circulating 25(OH)D concentrations exceeded 100–125 nmol/L [6–8]. Likewise, very recent data obtained in individuals receiving different daily doses of vitamin D confirmed a significantly increased risk of falls in patients achieving circulating 25(OH)D > 100 nmol/l [9]. Additionally, in patients with end-stage heart failure, a daily dose of 4000 IU vitamin D over a 3-year period resulted in a significantly increased risk of worsening disease, especially in the subgroup which achieved in-study 25(OH)D concentrations > 100 nmol/l [10].

Meanwhile, various trials have used daily vitamin D doses of 4000 IU. Such an intake also occurs in a minority of the general population, usually due to high-dose supplement use [11]. Since habitual vitamin D intake also contributes to total daily intake, we aimed to undertake a systematic review and meta-analysis on adverse events of long-term daily vitamin D supplementation of 3200–4000 IU. We focused our search on parameters of calcium metabolism, the musculo-skeletal system, and mortality.

## Methods

This meta-analysis was planned, conducted, and reported on the basis of a protocol that was developed in accordance with the PRISMA statement [12]. The protocol was registered at the PROSPERO international prospective register of systematic reviews as CRD42022349205.

### Eligibility criteria

Generally, only RCTs using daily vitamin D doses of 4000 IU for at least 6 months were eligible for inclusion. However, since dietary vitamin D intake also contributes to total daily vitamin D intake, and we aimed at evaluating safety of the current UL for vitamin D, we also accepted trials using daily supplemental vitamin D doses between 3200 and 4000 IU. We included only trials performed in age groups whose UL for vitamin D is 4000 IU, i.e., mean age 9 years or over. If calcium was given too, it had to be given to both study arms. Studies were excluded if they had fewer than ten participants in at least one arm or if the control arm received a vitamin D supplement of > 400 IU. Thus, the maximum allowance of supplemental daily vitamin D intake was 4400 IU (baseline vitamin D dose of 400 IU to both groups). Reporting of adverse events was a necessary condition for study eligibility, and we excluded RCTs which did not report these parameters separately for each study arm, with the sole exception that group-specific data presentation was not required for reported null effects. A null effect was only considered if the adverse event was explicitly stated. The following parameters were assessed: hypercalcemia, hypercalciuria, kidney stones, falls, fractures, hospitalization, and mortality. We applied no language or time restrictions, and there were no limitations with regard to patient characteristics or health status. Trials in pregnant women were also eligible for inclusion. The Population, Intervention, Comparison, Outcomes and Study (PICOS) criteria for inclusion or exclusion of studies are summarized in Table 1.

**Table 1:** PICOS criteria for inclusion or exclusion of studies

Parameters	Inclusion criteria	Exclusion criteria
Population	Human individuals	Mean age < 9 years
Intervention	Supplementation or food fortification with 3200 to 4000 IU vitamin D daily for at least 6 months	Non-daily administration, vitamin D dose < 3200 or > 4000 IU daily, vitamin D administration > 400 IU to the control group
Comparison	Adverse events by vitamin D vs. control	No reporting of adverse events
Outcome	Relative risk	No separate reporting of adverse events by study group
Study design	Only randomized controlled trials	No control group

*PICOS* population, intervention, comparison, outcome, study, *IU* international units

### Search strategy

We performed a systematic literature search for publications up to 31 October 2022 in several databases, such as PubMed, Web of Science, the Cochrane Library for reports, Google Scholar, and clinicaltrials.gov. The search terms are listed in Supplemental Table 1. We searched for the keywords in the titles and in the abstract, when available. Titles and abstracts of records identified in the primary search were screened, and all articles deemed potentially eligible for inclusion were retrieved in full-text format. Abstracts and unpublished results were not included. To identify additional papers, the reference lists of included studies and published reviews were also scanned. The search was performed independently by three researchers (AZ, CT, and VTS). Disagreements were resolved after debate by consensus.

### Data extraction

We performed data extraction with the use of a protocol designed before we conducted the data searches. The following information was extracted: year of publication, author, journal, country of origin, number of participants, percentage of females, mean age, study duration, vitamin D dose, type of control, health status, initial baseline 25(OH)D below 50 nmol/l, and number of study participants.

### Adverse events

The definition of adverse events was extracted from each article. With respect to hypercalcemia, it was assessed whether cut-offs of plasma calcium were provided in the article, or non-reported laboratory cut-offs were used for data reporting. For hypercalciuria assessment, we collected information about whether cases were based on elevated values in spot urine, fasting urine, or 24 h urine. Kidney stones, fractures, and falls were considered as stated in the articles. Pregnant women were assumed to be hospitalized if delivery became necessary in a specialized hospital, or postpartum hospitalization became necessary. All causes of death were eligible for inclusion in the mortality analysis.

### Data synthesis

We assessed the number of individuals with an adverse event in both the intervention and control groups. Data are presented as relative risk (RR) of the groups with their 95% confidence interval (CI). For data analysis, we used a fixed effects model, unless heterogeneity was proven. Heterogeneity was tested by the Chi-square test. The extent of between-study heterogeneity was also assessed by I^2^ statistics, thereby classifying 25%, 50%, and 75% as low, moderate, and high degrees of heterogeneity, respectively [13]. In studies that matched the inclusion criteria but reported zero events, the relative risk could not be calculated. To avoid overestimating the risk of hypercalcemia, these results were nevertheless added to the calculation of events per 1000 individuals. If more than one dosing regimen was used in the experimental arm (e.g., 3200 and 4000 IU), the number of participants in the control arm was divided by the number of experimental study arms. As with drug side effects, values between 1 and 10% were classified as frequent and values between 0.1 and 1% as occasionally.

### Data analysis

Several predefined subgroup analyses were performed where appropriate. To evaluate the effect of study duration, we conducted meta-analyses by trials with a duration ≤ 12 months and > 12 months. To explore the potential for a disease-related effect, meta-analyses were conducted according to heath status (healthy individuals vs. patients). Additional meta-analyses were performed by stratified analyses according to mean baseline 25(OH)D concentrations (< 50 nmol/l or ≥ 50 nmol/l), different control arms (placebo vs. low-dose vitamin D), age group (< 60 or ≥ 60 years), and number of participants in each study arm (> 100 and ≤ 100). To determine whether a statistically significant subgroup difference was detected, the test for subgroup differences from the Revman statistics program (see below) was used. All data for subgroup analyses were available from the original articles. Subgroup analysis was only performed if the number of included studies was ≥ 10 or displayed statistically significant heterogeneity. To investigate whether publication bias might affect the validity of the estimates, we constructed funnel plots of the regression of observed effect sizes against the corresponding SEs, weighted by the inverse of the pooled variance [14]. Study quality was assessed (independently by AZ and CT), by according to a tool provided by the Cochrane Handbook for Systematic Reviews of Interventions [15]. For the present meta-analysis, data were considered incomplete if results were not presented for both hypercalcemia and hypercalciuria risk. Additional risk of bias was assumed in case of infrequent or unsystematic data collection. For statistical significance, two-sided α was set at *P* < 0.05. All statistical analyses were conducted using RevMan (Review Manager. Version 5.3.: The Nordic Cochrane Centre. The Cochrane Collaboration. Copenhagen, 2014).

## Results

### Included studies

**Fig. 1:**
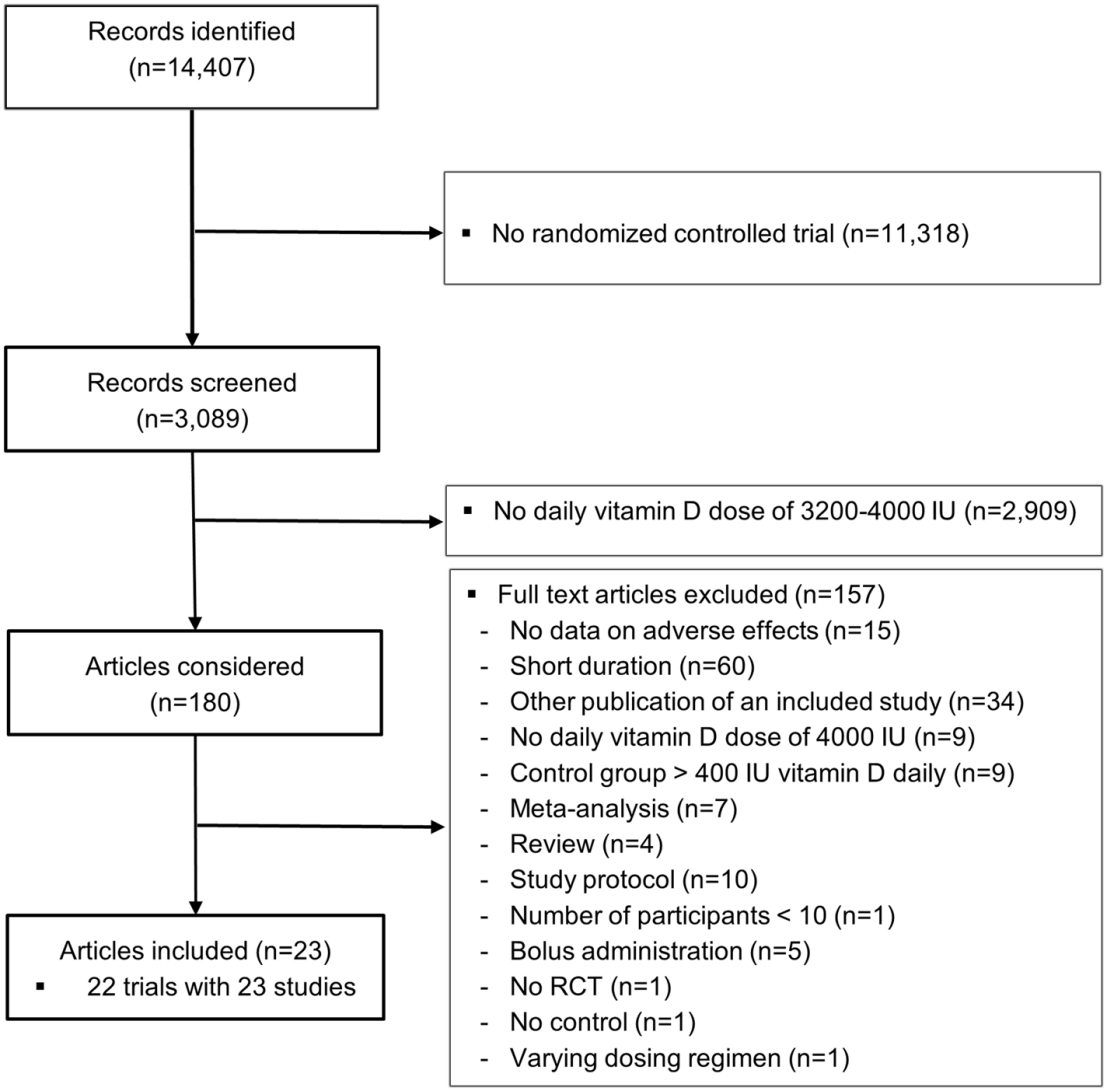
Flowchart of included and excluded articles

In total, we identified 14,407 abstracts (Fig. 1). We excluded 11,318 abstracts, because the studies were not randomized controlled trials, leaving 3089 records for screening. Of these, we excluded 2909 on the basis of screening titles and abstracts, because the vitamin D group did not receive 3200–4000 IU daily. Therefore, 180 articles were considered for systematic review by inspecting full-text articles. Of these, we excluded additional 157 articles for different reasons (Fig. 1). Thus, we could eventually include in our systematic review 23 articles on 22 trials [10, 16–38]. One trial using different dosing regimens included study arms with 3200 IU and 4000 IU vitamin D [17, 38]. Three other studies used a daily vitamin D supplement of 3200/3300 IU [34–36], whereas the remaining 18 trials used a daily vitamin D supplement of 4000 IU. Out of these 23 studies, 20 provided group-specific data on hypercalcemia, five on hypercalciuria, seven on kidney stones, four on falls, seven on hospitalization, and twelve on mortality. Since only one trial reported data on fracture risk, this outcome parameter was not included in our meta-analysis. Our search did not identify articles of interest for our review in languages other than English. All studies used supplemental vitamin D_3_. The characteristics of the studies are shown in Table 2. Excluded studies are listed in Supplemental Table 2. Almost all included studies were published after the IOM had released its last UL (4000 IU) in 2011. The trials comprised a total number of 12,952 individuals, 5,686 in the vitamin D arm and 7266 in the control arm. Mean baseline 25(OH)D values were < 50 nmol/l in 11 trials and ≥ 50 nmol/l in 11 trials. Of the 22 trials, 8 were performed in apparently healthy individuals with 3 studies including pregnant women, and 14 trials included different groups of patients. Mean age varied between 10 and 77 years. Of the 22 control groups, 16 received a placebo, one 200 IU vitamin D daily, and five 400 IU vitamin D daily. All but five groups in the vitamin D arm received 4000 IU supplemental vitamin D daily, three groups received 3200 IU, one 3300 IU, and one 4400 IU, where in the latter trial, both the vitamin D and control arm received in addition to the study medication of 4000 IU or placebo a vitamin D dose of 400 IU. Mean habitual vitamin D intake was reported in six trials only [17, 23, 30, 35, 37, 38], ranging from 50 [37] to 428 [35] IU daily. Three studies reported data on habitual supplement use [10, 34, 35], ranging from 0% [10] to 68% [35].

**Table 2:** List and characteristics of included trials

No.	References	Journal	Country of origin	Percent females	Mean age	Mean initial 25(OH)D < 50 nmol/l	Study duration	Vitamin D dose (IU)		Health status	Number of participants
								Verum	Placebo		
1	[37]	Am J Clin Nutr	Germany	67	48	Yes	12 mo	3300	0	Heart failure	165
2	[16]	J Bone Miner Res	USA	100	27	No	6 mo	4000	400	Healthy/pregnant	228
3	[17, 38]	Ann Intern Med	USA	100	67	Yes	12 mo	3200/4000	0	Healthy	60
4	[18]	BMJ Open	Sweden	73	53	No	12 mo	4000	0	Frequent ARI	124
5	[19]	Am J Clin Nutr	USA	50	14	Yes	6 mo	4000	0	Obese	44
6	[20]	JAMA	USA	35	40	Yes	6 mo	4000	0	Asthma	408
7	[21]	Circulation	USA	32	36	Yes	6 mo	4000	400	Prehypertension	363
8	[22]	Endocr Connect	India	100	22	No	6 mo	4000	0	PCOS	30
9	[23]	Clin Nutr	Mexico	100	57	No	6 mo	4000	0	Diabetes	104
10	[24]	J Am Coll Cardiol	UK	17	69	Yes	12 mo	4000	0	Heart failure	223
11	[25]	JAMA	USA	100	27	No	6 mo	4400	400	Healthy/pregnant	806
12	[10]	Eur Heart J	Germany	21	55	Yes	36 mo	4000	0	Heart failure	400
13	[26]	Osteoporos Int	UK	49	72	No	12 mo	4000	0	Healthy	194
14	[27]	Eur J Clin Microb Infect Dis	Sweden	57	41	Yes	12 mo	4000	0	MRSA carrier	65
15	[28]	New Engl J Med	USA	45	60	No	24 mo	4000	0	Prediabetes	2423
16	[29]	JAMA	USA	40	10	No	9 mo	4000	0	Asthma	192
17	[30]	J Clin Endocrinol Metab	Canada	51	62	No	36 mo	4000	400	Healthy	249
18	[31]	BMJ Nutr Prev Heath	Pakistan	100	26	Yes	6 mo	4000	400	Healthy/pregnant	235
19	[32, 33]	Am J Transplant J Bone Miner Res	Japan	31	52	Yes	12 mo	4000	0	Kidney transplanted	187
20	[34]	Ann Intern Med	USA	42	77	No	6 mo	4000	200	Healthy	325
21	[35]	Am J Clin Nutr	Finland	43	68	No	60 mo	3200	0	Healthy	1663
22	[36]	BMJ Open	UK	67	60	Yes	6 mo	3200	0	Respiratory tract infection	4464

*PCOS* polycystic ovarian syndrome, *ARI* acute respiratory tract infection

### Synthesis of results

**Fig. 2:**
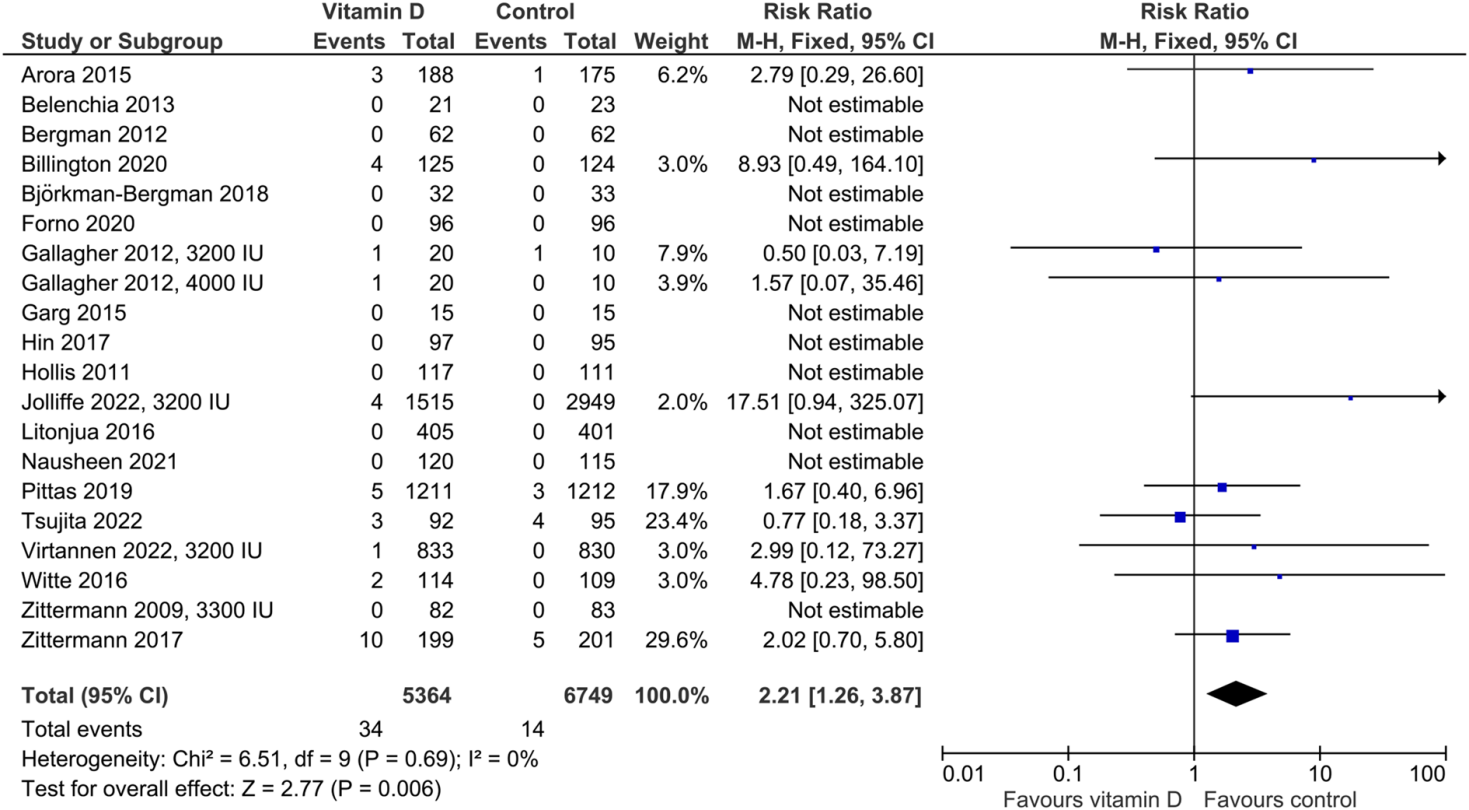
Effect of vitamin D on the risk of hypercalcemia. Data represent the relative risk of hypercalcemia in vitamin D vs. control with 95% confidence interval of individual studies and total effect. In the figure, the vitamin D dose is given if less than 4000 IU daily or different dosing regimens are used. In all other cases, 4000 IU vitamin D are supplemented. The x-axis indicates the relative risk, ranging from 0.01 to 100. Values < 1 favour vitamin D and values > 1 favour control

The synthesis of the risk of hypercalcemia is presented in Fig. 2. Out of the 20 studies which provided data on hypercalcemia risk, ten studies reported no case of hypercalcemia, neither in the vitamin D group nor in the control group. Cut-offs of plasma calcium for hypercalcemia were 2.55 mmol/l in four trials, 2.60 mmol/l in two trials, 2.65 in one trial, 2.70 in one trial, and 2.75 mmol/l in one trial, and were not presented in ten trials. In the ten studies that reported at least one case of hypercalcemia, vitamin D supplementation resulted in a significantly higher risk of hypercalcemia with an RR of 2.21 (95%CI: 1.26–3.87). Altogether, the frequency of hypercalcemia in the control and vitamin D group was 0.21% (14 in 6749 individuals) and 0.63% (34 in 5364 individuals), respectively, resulting in a vitamin D-induced frequency of hypercalcemia of 0.42% or 4 cases per 1000 individuals. In our meta-analysis, the *P* and *I*^2^ values of 0.69 and 0%, respectively, indicate the absence of heterogeneity. In line with this, subgroup analysis of vitamin D vs. controls did not result in significant differences in hypercalcemia risk with respect to study duration, baseline 25(OH)D, heath status, age at enrollment, and type of control arm (Supplemental Fig. 1). However, subgroup analysis revealed borderline significance in trials with > 100 and ≤ 100 study participants with an RR of 2.63 (95%CI: 1.30–5.30; 7 studies) and 0.80 (95%CI: 0.24–2.62; 3 studies), respectively (P_interaction_ = 0.06). All ten studies that reported at least one case of hypercalcemia achieved mean in-study 25(OH)D concentration between 103 and 130 nmol/l, with the exception of the study by Arora et al. [20], which only achieved 83 nmol/l.

**Fig. 3:**
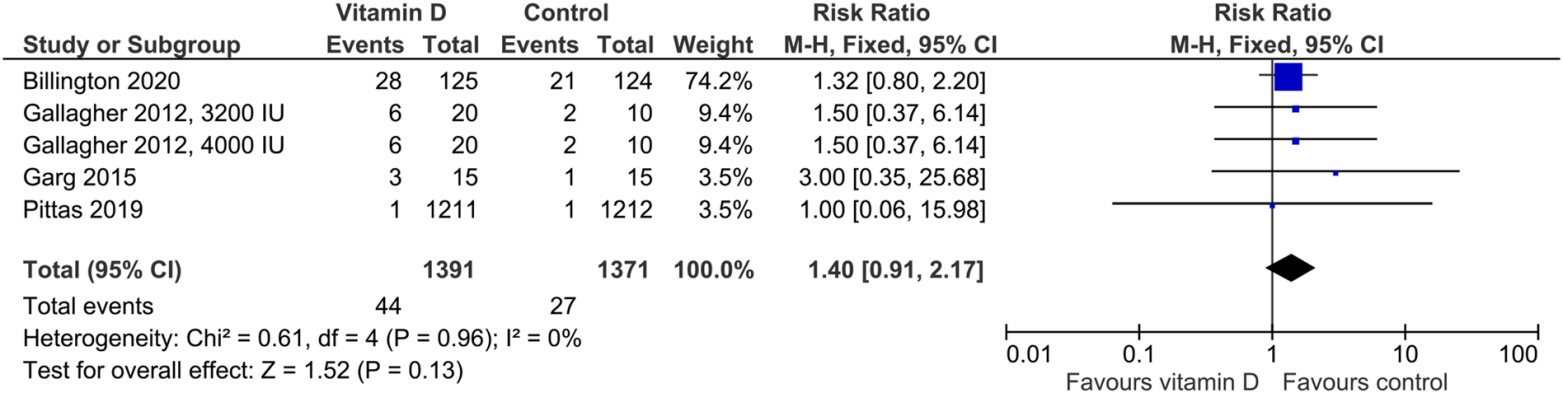
Effect of vitamin D on the risk of hypercalciuria. Data represent the relative risk of hypercalciuria in vitamin D vs. control with 95% confidence interval of individual studies and total effect. In the figure, the vitamin D dose is given if less than 4000 IU daily or different dosing regimens are used. In all other cases, 4000 IU vitamin D are supplemented. The x-axis indicates the relative risk, ranging from 0.01 to 100. Values < 1 favour vitamin D and values > 1 favour control

**Fig. 4:**
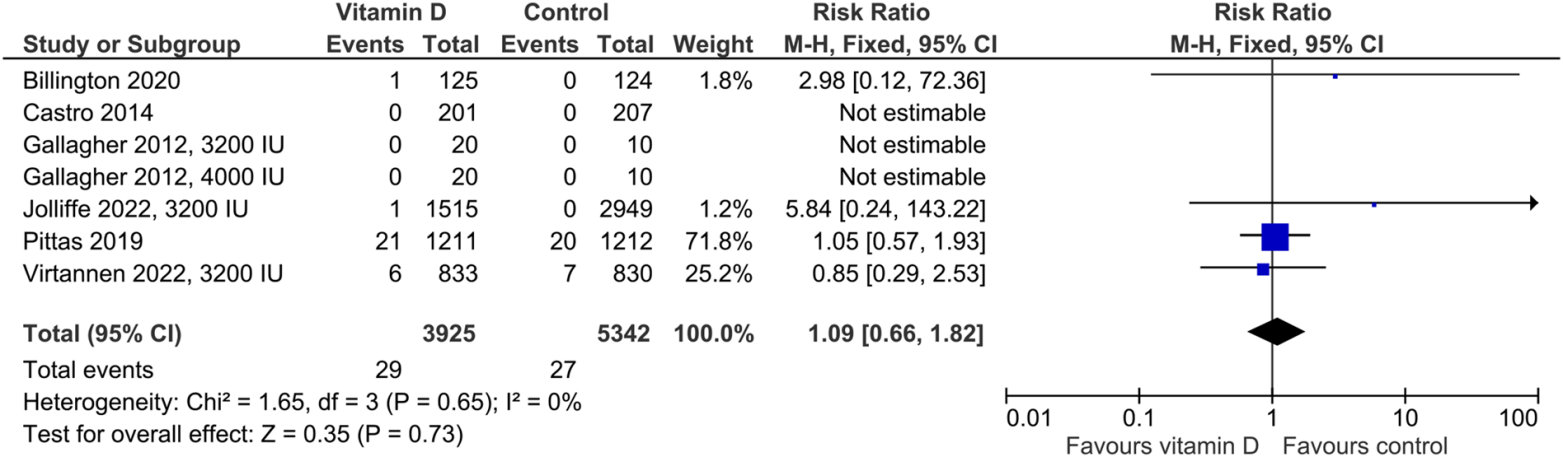
Effect of vitamin D on the risk of kidney stones. Data represent the relative risk of kidney stones in vitamin D vs. control with 95% confidence interval of individual studies and total effect. In the figure, the vitamin D dose is given if less than 4000 IU daily or different dosing regimens are used. In all other cases, 4000 IU vitamin D are supplemented. The x-axis indicates the relative risk, ranging from 0.01 to 100. Values < 1 favour vitamin D and values > 1 favour control

**Fig. 5:**
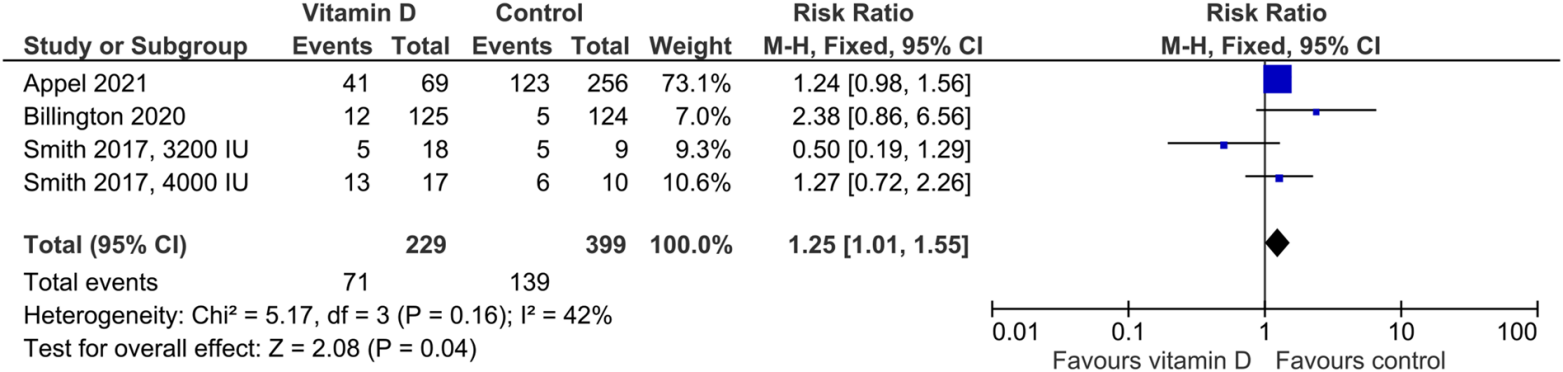
Effect of vitamin D on the risk of falls. Data represent the relative risk of falls in vitamin D vs. control with 95% confidence interval of individual studies and total effect. In the figure, the vitamin D dose is given if less than 4000 IU daily or different dosing regimens are used. In all other cases, 4000 IU vitamin D are supplemented. The x-axis indicates the relative risk, ranging from 0.01 to 100. Values < 1 favour vitamin D and values > 1 favour control

**Fig. 6:**
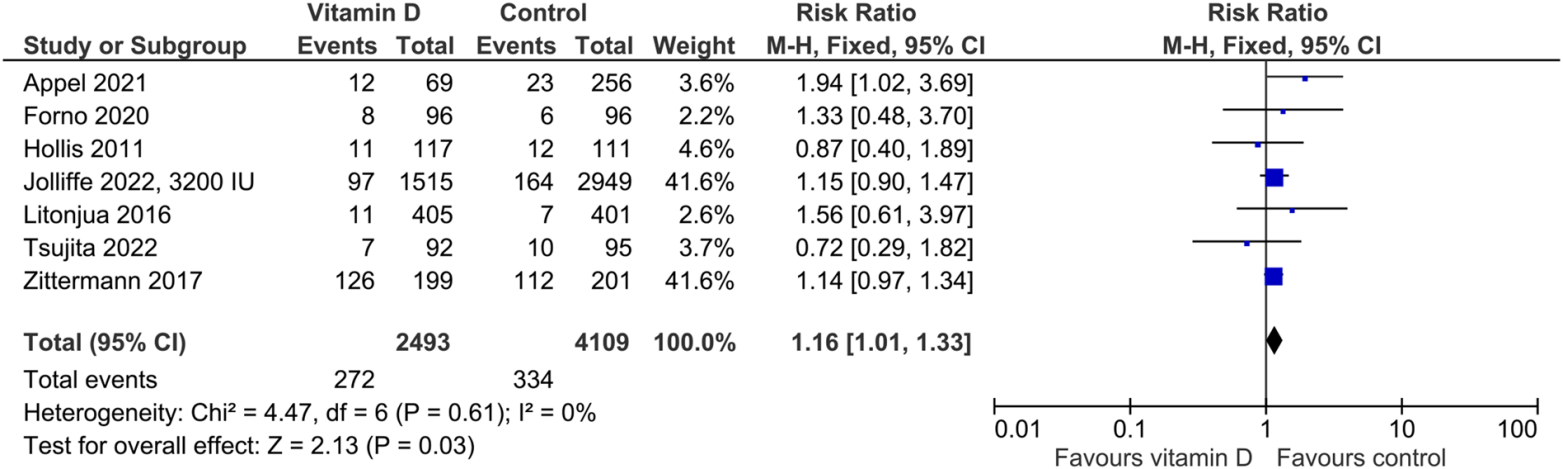
Effect of vitamin D on the risk of hospitalization. Data represent the relative risk of hospitalization in vitamin D vs. control with 95% confidence interval of individual studies and total effect. In the figure, the vitamin D dose is given if less than 4000 IU daily or different dosing regimens are used. In all other cases, 4000 IU vitamin D are supplemented. The x-axis indicates the relative risk, ranging from 0.01 to 100. Values < 1 favour vitamin D and values > 1 favour control

**Fig. 7:**
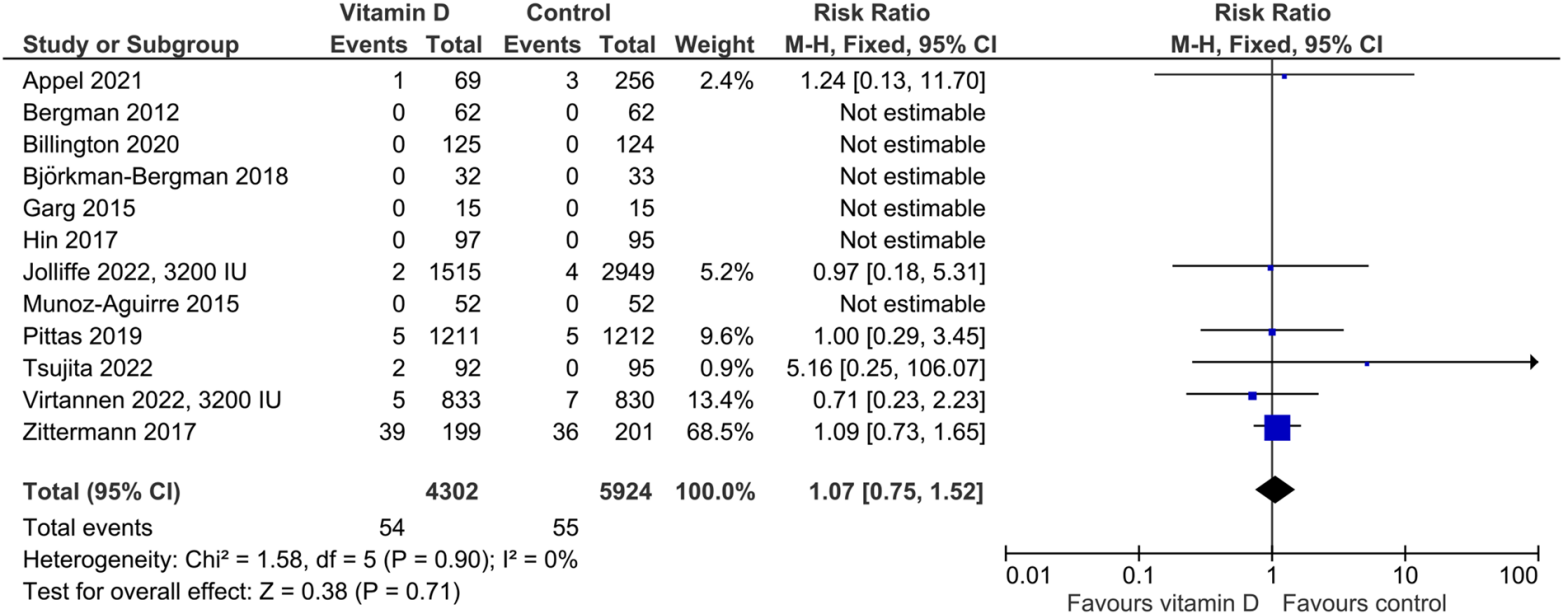
Effect of vitamin D on the risk of mortality. Data represent the relative risk of mortality in vitamin D vs. control with 95% confidence interval of individual studies and total effect. In the figure, the vitamin D dose is given if less than 4000 IU daily or different dosing regimens are used. In all other cases, 4000 IU vitamin D are supplemented. The x-axis indicates the relative risk, ranging from 0.01 to 100. Values < 1 favour vitamin D and values > 1 favour control

In the five studies providing data on hypercalciuria, the risk was non-significantly higher in vitamin D-supplemented individuals than in controls (Fig. 3) with an RR of 1.40 (95%CI: 0.91–2.17). Cut-offs for hypercalciuria were based on 24 h urinary calcium > 7.5 mmol/l, > 7.5 mmol/day if body weight was ≤ 75 kg or > 0.1 mmol/kg body weight/day if body weight was > 75 kg, > 0.1 mmol/kg body weight/day, and fasting urinary calcium/creatinine ratio > 0.375 in one trial each. Four trials did not provide group-specific data on hypercalciuria, although the number of cases was greater than zero. Regarding kidney stones, the RR of 1.09 (95%CI: 0.66–1.82) was similar for the vitamin D vs. control group (Fig. 4). Four studies reported data on the risk of falls (Fig. 5) with a significantly higher risk in the vitamin D-supplemented vs. the control group (RR 1.25, 95%CI: 1.01–1.25). Vitamin D supplementation also increased the risk of hospitalization (Fig. 6) with an RR of 1.16 (95%CI: 1.01–1.33). Mortality risk was similar in the vitamin D and control groups (Fig. 7) with an RR of 1.07 (95%CI: 0.75–1.52). According to the Chi-square test, there was no statistically significant evidence of heterogeneity for risk of hypercalciuria, falls, hospitalization, or death (Figs. 3–7). However, the I^2^ value of 42% indicates moderate heterogeneity regarding the risk of falls. In the four studies which were included in the meta-analysis on falls and the seven studies which were included in the meta-analysis on hospitalization, the vitamin D-induced frequency was 2.7% or 27 cases per 1000 individuals and 3.0% or 30 cases per 1000 individuals, respectively.

### Sensitivity analysis

In sensitivity analyses, we excluded the trial by Zittermann et al. [9], because of an extraordinary high number of adverse events. However, results did not change substantially regarding the RR for hypercalcemia (2.29; 95%CI: 1.18–4.43), hospitalization (1.18, 95%CI: 0.96–1.44), or death (1.02; 95%CI: 0.52–2.00). Likewise, exclusion of the study arm of 3200 IU by Smith et al. [36] did not change hospitalization risk substantially (1.33, 95%CI: 1.07–1.66).

### Publication bias and study quality

Inspection of the funnel plots of included trials did not provide evidence of publication bias for any of the outcomes considered (Supplementary Figs. 2–7). Regarding study quality, results are presented in Supplementary Fig. 8. In the majority of trials, risk of incomplete assessment of safety-related outcomes was high.

## Discussion

Our meta-analysis indicates that compared with placebo or low-dose (400 IU/d) vitamin D, a supplement of 3200–4000 IU vitamin D daily results in significantly higher risks of hypercalcemia (RR = 2.21, 95%CI: 1.26–3.87), falls (RR = 1.25, 95%CI: 1.01–1.25), and hospitalization (RR = 1.16, 95%CI: 1.01–1.33). However, data also indicate that the risk of kidney stones and mortality is not significantly affected by a daily vitamin D dose of 4000 IU. Nevertheless, it is also noteworthy that in many trials, the quality of reporting adverse events was poor and several trials did not address the risk of important adverse events at all.

Since in our meta-analysis, 4 cases of hypercalcemia per 1000 individuals were vitamin D-induced, hypercalcemia at a daily dose of 3200–4000 IU has to be considered an occasional adverse event. In 2011, the IOM stated that the toxicity of hypercalcemia becomes evident at vitamin D intakes above 25,000 IU/day, corresponding to a serum 25OHD level of about 500 nmol/L [2]. This assumption is in general agreement with our finding that > 99.5% of study participants did not develop vitamin D-induced hypercalcemia during administration of 3200–4000 IU daily. Four earlier meta-analyses reported data of hypercalcemia risk from vitamin D supplementation trials [5, 39–41], with mean relative risks being 36% [39], 57% [40], 54% [41], and 93% [5] higher than in controls. Only the meta-analysis with the highest number of included trials (n = 37) reported significantly higher hypercalcemia risk by vitamin D supplementation [41], whereas the meta-analysis with the highest RR of hypercalcemia for the vitamin D group [5] reported only borderline significance, based on 10 trials. In both meta-analyses [5, 41], more than 50% of the included trials used intermittent high vitamin D doses or daily doses beyond 4000 IU. Therefore, it is an important finding of the present meta-analysis that daily supplemental doses of 3200–4000 IU vitamin D increase hypercalcemia risk slightly, yet significantly. The vast majority of trials reporting cases of hypercalcemia achieved mean in-study 25(OH)D concentrations at the upper end of the range, which is still considered adequate (100–125 nmol/l). However, our data support the assumption that this upper range may be not completely safe, but individual participant data meta-analyses would be required to evaluate the safety of certain 25(OH)D cut-offs. Subgroup analysis indicates that small studies obviously underestimated the risk of hypercalcemia. Because of the small number of included small studies in our subgroup analysis and in accordance with the Cochrane Manual for Meta-Analyses, a borderline significant result such as our P value of 0.06, instead of the conventional value of 0.05, can be considered statistically significant [42]. In ten other studies, most of which also were small studies (< 100 participants per study arm), the hypercalcemia incidence of 4/1000 was probably too low to cause any case of hypercalcemia. The situation seems to be similar to that of drugs, where occasional or rare adverse events are often not detected until phase IV (post-marketing) studies, based on large datasets, are performed. Therefore, further large studies may clarify the dose–response effect of vitamin D on the risk of hypercalcemia. Another issue is that usually fasting blood samples are used to measure plasma calcium concentrations. The IOM has stated that hypercalcemia is the consequence of increased calcium resorption from bone [2], which would also translate into elevated fasting plasma calcium concentrations. However, 4000 IU vitamin D daily may rarely induce toxic effects on bone, but may increase prandial or postprandial serum calcium more pronouncedly than fasting plasma calcium, since 4000 IU vitamin D increases intestinal calcium absorption rate significantly (by about 6–7%) [43]. Therefore, the real effect of 4000 IU vitamin D on plasma calcium may be underestimated when measuring fasting calcium levels. In line with this assumption, some studies also reported a significant mean in-study increase in plasma calcium in the vitamin D arm without exceeding the cut-off of hypercalcemia [10, 26, 44]. The clinical importance of small elevations in serum calcium is highlighted by findings that heart failure incidence increases progressively from a serum calcium of 2.25 mmol/L up to 2.75 mmol/L [45] and genetically predicted lifelong higher concentrations of serum calcium may shorten life expectancy and increase cardiovascular disease (CVD) risk [46].

It has long been known that the higher 25(OH)D values observed in summer when compared to winter are also associated with significantly higher urinary calcium excretion, despite similar dietary calcium intakes in both seasons [47]. Unfortunately, many vitamin D trials do not routinely collect urine samples. This can explain the smaller number of reports on hypercalciuria than on hypercalcemia in earlier meta-analyses [5, 41] and also in our meta-analysis. Generally, the non-significantly higher risk of hypercalciuria in the vitamin D arm of our analysis is in line with earlier data of an increased risk of hypercalciuria by vitamin D supplementation [5, 41]. From a pathophysiological point of view, it appears logical that, in the event of vitamin D toxicity, hypercalcemia is preceded by hypercalciuria. Nevertheless, data also indicate that even 4000 IU daily are safe regarding kidney stones. Although high 25(OH)D levels must be considered as a risk factor for kidney stones [48], the etiology of kidney stones is complex. High fluid intake and consumption of fruits and foods high in fiber are important preventive factors [49, 50]. Notably, urinary calcium excretion is also strongly and positively related to urinary sodium excretion, and thus with dietary salt intake [51]. As supported by our results, there seems to be no simple association between vitamin D status and risk of kidney stones.

The higher risk of falls in the vitamin D-supplemented study participants should be discussed with caution, because results are based on a few trials only. However, our analysis indicates this risk to be a frequent complication. Results are in line with results indicating an increased risk of falls at circulating 25(OH)D of 100 nmol/l and above [6, 9]. We can only speculate that higher plasma calcium concentrations in patients receiving 4000 IU daily than in controls, as reported in some studies [10, 26], may also affect neuromuscular activity and thus the risk of falls. The higher risk of hospitalization in the vitamin D group may at least in part be the result of a higher risk of adverse events, such as falls or CVD worsening [6, 9, 10]. Obviously, however, this did not translate into an increased mortality, probably because some of the causes for hospitalization were not life-threatening or because of the high quality of the health system in high-income countries, being able to prevent premature death in many cases. Although the UL concept has been developed for the general population, non-classical safety-related outcomes of vitamin D usually related to diseased individuals, such as falls, hospitalization, and death, should also be considered in future trials, given the high percentage of older and multimorbid individuals in aging societies. We are well aware that our meta-analysis was not designed to assess potential benefits of moderately high-dose vitamin D supplementation, but the overall adverse effects regarding some clinical outcomes strongly argue against the use of such vitamin D dosages in clinical routine. We do, however, acknowledge that certain patient characteristics might justify such high vitamin D doses as, e.g., in patients with malabsorption and/or osteomalacia, but in such rare cases, a frequent monitoring of 25(OH)D concentrations and parameters of calcium metabolism is strongly advised.

Our analysis has some limitations. First, a major issue is the incomplete reporting of adverse events in many included trials. Since hypercalcemia is considered the general indicator of vitamin D toxicity, other potential adverse events such as the risk of falls and hospitalization may have been underestimated to date. However, it is also noteworthy that falls were exclusively reported in studies of individuals with a mean age of 60 years or older [29, 33, 37]. Hospitalizations were only reported in pregnant women [15, 24], diseased individuals [28, 31, 36], and elderly people [33]. For healthy and younger males or non-pregnant women, and thus for an important target group of the UL, these adverse events may not be relevant. Second, data availability was particularly low for the risk of falls and fractures, which may at least in part be explained by the inclusion of healthy, young, and middle-aged, and male individuals. Even with respect to hypercalcemia, however, several trials did not address this issue at all. Third, different cut-offs have been used for characterization of hypercalcemia, ranging from 2.55 to 2.75 mmol/l. This can have a profound effect on the frequency of reported cases of hypercalcemia and may, in case of low cut-offs, mask causal associations of supplemental vitamin D with CVD or kidney diseases. It is also a drawback that we used aggregate data and not individual participant data for our meta-analyses. Finally, in all trials using 4000 IU vitamin D daily, the UL was slightly exceeded because of additional, but unreported habitual vitamin D intake by foods and supplements, and this has to be considered.

In conclusion, supplemental daily vitamin D doses of 3200–4000 IU appear to increase the risk of hypercalcemia and some other adverse events in a small proportion of individuals, indicating that this dose is not completely safe. Thus, more than a decade after publication of the 2011 IOM report uncertainty regarding the safety of a daily dose of 4000 IU still remains. Our data indicate that, similar to phase IV drug studies, large numbers of individuals need to be studied to capture occasional or even rare adverse events. Therefore, there is an urgent need for a rigorous reporting of safety-related outcomes in vitamin D supplementation or fortification trials, at least if moderately high vitamin D doses are used. This is all the more important as during recent years, the intake of 4000 IU daily has continuously increased [11], and some nonofficial organizations recommend an intake of even 5000 IU vitamin D daily and more [52]. Finally, regarding the risks of falls and hospitalization, UL values, which are considered for the healthy general population, should not be adopted a priori in the clinical setting.

## Supplementary Information

Below is the link to the electronic supplementary material.

Supplementary file1 (DOCX 127 KB)

## Data Availability

Data are available on reasonable request.
