# Supplementary material for: Long-term supplementation with 3200 to 4000 IU of vitamin D daily and adverse events: a systematic review and meta-analysis of randomized controlled trials
Source: Eur J Nutr. 2023 Feb 28;62(4):1833–44. doi: 10.1007/s00394-023-03124-w (PMC10195747; doi:10.1007/s00394-023-03124-w)

Supplementary Information

**Long-term supplementation with 3200 to 4000 IU of vitamin D daily and adverse events: a systematic review and meta-analysis of randomized controlled trials**

Zittermann A et al.

Page

Supplemental Table 1: Search strategy 2

Supplemental Table 2: List of excluded articles 3

Supplemental Figure 1: Subgroup analyses of vitamin D vs. control on the

relative risk of hypercalcemia 19

Supplemental Figure 2: Funnel plot of included studies for the assessment

of the risk of hypercalcemia 20

Supplemental Figure 3: Funnel plot of included studies for the assessment

of the risk of hypercalciuria 21

Supplemental Figure 4: Funnel plot of included studies for the assessment

of the risk of kidney stones 22

Supplemental Figure 5: Funnel plot of included studies for the assessment

of the risk of falls 23

Supplemental Figure 6: Funnel plot of included studies for the assessment

of the risk of hospitalization 24

Supplemental Figure 7: Funnel plot of included studies for the assessment

of the risk of mortality 25

Supplemental Figure 8: Methodological quality graph: authors´ judgments

about each methodological quality item regarding

adverse events presented as percentages for

included studies 26

**Supplemental Table 1:** Search strategy

| Study type | Randomized controlled trial OR randomised controlled trial OR RCT |
| --- | --- |
| Vitamin D | vitamin D OR cholecalciferol OR ergocalciferol OR vitamin D_3_ OR vitamin D_2_ |
| Dose | 4000 IU OR 3200 IU OR 3300 IU OR 3400 IU OR 3500 IU OR 3600 IU OR 3700 IU OR 3800 IU or 100 µg OR 80 µg OR 83 µg OR 85 µg OR 88 µg OR 90 µg OR 93 µg OR 95 µg |
| Type of intake | supplementation OR supplement OR administration OR use OR food fortification OR food enrichment |
| Adverse event | Hypercalcemia OR hypercalcaemia OR hypercalciuria OR kidney stones OR kidney stone OR fracture OR fractures OR falls OR hospitalization OR hospitalisation OR morbidity OR death OR mortality |

**Supplemental Table 2:** List of excluded articles

| 1 | Jolliffe DA, Vivaldi G, Chambers ES, Cai W, Li W, Faustini SE, Gibbons JM, Pade C, Coussens AK, Richter AG, McKnight Á, Martineau AR.Vitamin D Supplementation Does Not Influence SARS-CoV-2 Vaccine Efficacy or Immunogenicity: Sub-Studies Nested within the CORONAVIT Randomised Controlled Trial. Nutrients. 2022 Sep 16;14(18):3821. | Other publication of an included study |
| --- | --- | --- |
| 2 | Ahmad Fuzi SF, Su Peng L, Zabaha Zalbahar N, Ab Manan N, Mohamad Alwi MN. Effect of vitamin D3-fortified fruit juice supplementation of 4000 IU daily on the recovery of iron status in childbearing-aged women with marginally low iron stores: Protocol for an 8-week, parallel group, double-blind randomized controlled trial. PLoS One. 2022 Mar 25;17(3):e0265772. | Short duration |
| 3 | Dong Y, Chen L, Huang Y, Raed A, Havens R, Dong Y, Zhu H. Sixteen-Week Vitamin D3 Supplementation Increases Peripheral T Cells in Overweight Black Individuals: Post hoc Analysis of a Randomized, Double-Blinded, Placebo-Controlled Trial. Nutrients. 2022 Sep 22;14(19):3922. | Short duration |
| 4 | De Niet S, Trémège M, Coffiner M, Rousseau AF, Calmes D, Frix AN, Gester F, Delvaux M, Dive AF, Guglielmi E, Henket M, Staderoli A, Maesen D, Louis R, Guiot J, Cavalier E. Positive Effects of Vitamin D Supplementation in Patients Hospitalized for COVID-19: A Randomized, Double-Blind, Placebo-Controlled Trial. Nutrients. 2022 Jul 26;14(15):3048. | Dosing regimen does not meet inclusion criteria |
| 5 | Woo JS, Woo Y, Jang JY, Ha SJ. Effect of vitamin D on endothelial and ventricular function in chronic heart failure patients: A prospective, randomized, placebo-controlled trial. Medicine (Baltimore). 2022 Jul 22;101(29):e29623. | No safety data presented |
| 6 | Desouza C, Chatterjee R, Vickery EM, Nelson J, Johnson KC, Kashyap SR, Lewis MR, Margolis K, Pratley R, Rasouli N, Sheehan PR, Pittas AG; D2d Research Group.The effect of vitamin D supplementation on cardiovascular risk in patients with prediabetes: A secondary analysis of the D2d study.  J Diabetes Complications. 2022 Aug;36(8):108230. | Other publication of an included study |
| 7 | Chen Y, Liang Y, Guo H, Meng K, Qiu J, Benardot D. Muscle-Related Effect of Whey Protein and Vitamin D3 Supplementation Provided before or after Bedtime in Males Undergoing Resistance Training. Nutrients. 2022 May 30;14(11):2289. | Short duration |
| 8 | Hew-Butler T, Aprik C, Byrd B, Sabourin J, VanSumeren M, Smith-Hale V, Blow A.Vitamin D supplementation and body composition changes in collegiate basketball players: a 12-week randomized control trial. J Int Soc Sports Nutr. 2022 Mar 22;19(1):34-48. | Short duration |
| 9 | Muhihi A, Fawzi WW, Aboud S, Nagu TJ, Ulenga N, Wang M, Mugusi F, Sudfeld CR. Cholecalciferol Supplementation Does Not Affect the Risk of HIV Progression, Viral Suppression, Comorbidities, Weight Loss, and Depression among Tanzanian Adults Initiating Antiretroviral Therapy: Secondary Outcomes of a Randomized Trial. J Nutr. 2022 Aug 9;152(8):1983-1990. | Bolus administered |
| 10 | Cai Y, Wanigatunga AA, Mitchell CM, Urbanek JK, Miller ER 3rd, Juraschek SP, Michos ED, Kalyani RR, Roth DL, Appel LJ, Schrack JA. The effects of vitamin D supplementation on frailty in older adults at risk for falls.  BMC Geriatr. 2022 Apr 10;22(1):312. | Other publication of an included study |
| 11 | Naderi M, Kordestani H, Sahebi Z, Khedmati Zare V, Amani-Shalamzari S, Kaviani M, Wiskemann J, Molanouri Shamsi M.Serum and gene expression profile of cytokines following combination of yoga training and vitamin D supplementation in breast cancer survivors: a randomized controlled trial.  BMC Womens Health. 2022 Mar 24;22(1):90. | Short duration |
| 12 | Helde Frankling M, Klasson C, Björkhem-Bergman L. 25-Hydroxyvitamin D in Cancer Patients Admitted to Palliative Care: A Post-Hoc Analysis of the Swedish Trial 'Palliative-D'. Nutrients. 2022 Jan 29;14(3):602. | Short duration |
| 13 | Johnson KC, Pittas AG, Margolis KL, Peters AL, Phillips LS, Vickery EM, Nelson J, Sheehan PR, Reboussin D, Malozowski S, Chatterjee R; D2d research group. Safety and tolerability of high-dose daily vitamin D3 supplementation in the vitamin D and type 2 diabetes (D2d) study-a randomized trial in persons with prediabetes. Eur J Clin Nutr. 2022 Aug;76(8):1117-1124. | Other publication of an included study |
| 14 | Tsujita M, Doi Y, Obi Y, Hamano T, Tomosugi T, Futamura K, Okada M, Hiramitsu T, Goto N, Isaka Y, Takeda A, Narumi S, Watarai Y. Cholecalciferol Supplementation Attenuates Bone Loss in Incident Kidney Transplant Recipients: A Prespecified Secondary Endpoint Analysis of a Randomized Controlled Trial. J Bone Miner Res. 2022 Feb;37(2):303-311. | Other publication of an included study |
| 15 | Maghsoumi-Norouzabad L, Zare Javid A, Mansoori A, Dadfar M, Serajian A. Vitamin D3 Supplementation Effects on Spermatogram and Oxidative Stress Biomarkers in Asthenozoospermia Infertile Men: a Randomized, Triple-Blind, Placebo-Controlled Clinical Trial. Reprod Sci. 2022 Mar;29(3):823-835. | Short duration |
| 16 | Rasouli N, Brodsky IG, Chatterjee R, Kim SH, Pratley RE, Staten MA, Pittas AG; D2d Research Group. Effects of Vitamin D Supplementation on Insulin Sensitivity and Secretion in Prediabetes. J Clin Endocrinol Metab. 2022 Jan 1;107(1):230-240. | Other publication of an included study |
| 17 | Nadeem S, Tangpricha V, Ziegler TR, Rhodes JE, Leong T, Xiang Y, Greenbaum LA. Randomized trial of two maintenance doses of vitamin D in children with chronic kidney disease. Pediatr Nephrol. 2022 Feb;37(2):415-422. | Control group supplemented with > 400 IU |
| 18 | Rosser FJ, Han YY, Forno E, Bacharier LB, Phipatanakul W, Guilbert TW, Cabana MD, Ross K, Blatter J, Durrani S, Luther J, Wisniewski SR, Celedón JC. Effect of vitamin D supplementation on total and allergen-specific IgE in children with asthma and low vitamin D levels. J Allergy Clin Immunol. 2022 Jan;149(1):440-444.e2. | Other publication of an included study |
| 19 | Ahmad Fuzi SF, Su Peng L, Zabaha Zalbahar N, Ab Manan N, Mohamad Alwi MN. Effect of vitamin D3-fortified fruit juice supplementation of 4000 IU daily on the recovery of iron status in childbearing-aged women with marginally low iron stores: Protocol for an 8-week, parallel group, double-blind randomized controlled trial. PLoS One. 2022 Mar 25;17(3):e0265772. doi: 10.1371/journal.pone.0265772. eCollection 2022 | Short duration |
| 20 | Chen Y, Liang Y, Guo H, Meng K, Qiu J, Benardot D. Muscle-Related Effect of Whey Protein and Vitamin D3 Supplementation Provided before or after Bedtime in Males Undergoing Resistance Training. Nutrients. 2022 May 30;14(11):2289. doi: 10.3390/nu14112289. PMID: 35684089 Free PMC article. Clinical Trial. | Short duration |
| 21 | Hew-Butler T, Aprik C, Byrd B, Sabourin J, VanSumeren M, Smith-Hale V, Blow A. Vitamin D supplementation and body composition changes in collegiate basketball players: a 12-week randomized control trial. J Int Soc Sports Nutr. 2022 Mar 22;19(1):34-48. doi: 10.1080/15502783.2022.2046444. eCollection 2022. | Short duration |
| 22 | Cai Y, Wanigatunga AA, Mitchell CM, Urbanek JK, Miller ER 3rd, Juraschek SP, Michos ED, Kalyani RR, Roth DL, Appel LJ, Schrack JA. The effects of vitamin D supplementation on frailty in older adults at risk for falls. BMC Geriatr. 2022 Apr 10;22(1):312. doi: 10.1186/s12877-022-02888-w. | Other publication of an included study |
| 23 | Ahmad Fuzi SF, Su Peng L, Zabaha Zalbahar N, Ab Manan N, Mohamad Alwi MN. Effect of vitamin D3-fortified fruit juice supplementation of 4000 IU daily on the recovery of iron status in childbearing-aged women with marginally low iron stores: Protocol for an 8-week, parallel group, double-blind randomized controlled trial. PLoS One. 2022 Mar 25;17(3):e0265772. doi: 10.1371/journal.pone.0265772. eCollection 2022. | Study protocol |
| 24 | Naderi M, Kordestani H, Sahebi Z, Khedmati Zare V, Amani-Shalamzari S, Kaviani M, Wiskemann J, Molanouri Shamsi M. Serum and gene expression profile of cytokines following combination of yoga training and vitamin D supplementation in breast cancer survivors: a randomized controlled trial. BMC Womens Health. 2022 Mar 24;22(1):90. doi: 10.1186/s12905-022-01671-8. | Short duration |
| 25 | Helde Frankling M, Klasson C, Björkhem-Bergman L. 25-Hydroxyvitamin D in Cancer Patients Admitted 8to Palliative Care: A Post-Hoc Analysis of the S9wedish Trial 'Palliative-D'. Nutrients. 2022 Jan 2910;14(3):602. doi: 10.3390/nu14030602. | Short duration |
| 26 | Wang R, DeGruttola V, Lei Q, Mayer KH, Redline S, Hazra A, Mora S, Willett WC, Ganmaa D, Manson JE. The vitamin D for COVID-19 (VIVID) trial: A pragmatic cluster-randomized design. Contemp Clin Trials. 2021 Jan;100:106176. | Study protocol |
| 27 | Nowak JK, Sobkowiak P, Drzymała-Czyż S, Krzyżanowska-Jankowska P, Sapiejka E, Skorupa W, Pogorzelski A, Nowicka A, Wojsyk-Banaszak I, Kurek S, Zielińska-Psuja B, Lisowska A, Walkowiak J. Fat-Soluble Vitamin Supplementation Using Liposomes, Cyclodextrins, or Medium-Chain Triglycerides in Cystic Fibrosis: A Randomized Controlled Trial. Nutrients. 2021 Dec 20;13(12):4554. doi: 10.3390/nu13124554. | Short duration |
| 28 | Guralnik JM, Sternberg AL, Mitchell CM, Blackford AL, Schrack J, Wanigatunga AA, Michos E, Juraschek SP, Szanton S, Kalyani R, Cai Y, Appel LJ; STURDY Collaborative Research Group. Effects of Vitamin D on Physical Function: Results from the STURDY Trial. J Gerontol A Biol Sci Med Sci. 2021 Dec 20:glab379. doi: 10.1093/gerona/glab379. Online ahead of print. | Other publication of an included study |
| 29 | Maghsoumi-Norouzabad L, Zare Javid A, Mansoori A, Dadfar M, Serajian A. Vitamin D3 Supplementation Effects on Spermatogram and Oxidative Stress Biomarkers in Asthenozoospermia Infertile Men: a Randomized, Triple-Blind, Placebo-Controlled Clinical Trial. Reprod Sci. 2022 Mar;29(3):823-835. doi: 10.1007/s43032-021-00769-y. Epub 2021 Oct 18. | Short duration |
| 30 | Rasouli N, Brodsky IG, Chatterjee R, Kim SH, Pratley RE, Staten MA, Pittas AG; D2d Research Group. Effects of Vitamin D Supplementation on Insulin Sensitivity and Secretion in Prediabetes. J Clin Endocrinol Metab. 2022 Jan 1;107(1):230-240. doi: 10.1210/clinem/dgab649. | Other publication of an included study |
| 31 | Nadeem S, Tangpricha V, Ziegler TR, Rhodes JE, Leong T, Xiang Y, Greenbaum LA. Randomized trial of two maintenance doses of vitamin D in children with chronic kidney disease. Pediatr Nephrol. 2022 Feb;37(2):415-422. doi: 10.1007/s00467-021-05228-z. Epub 2021 Aug 15. | No low-dose vitamin D control |
| 32 | Kim SH, Brodsky IG, Chatterjee R, Kashyap SR, Knowler WC, Liao E, Nelson J, Pratley R, Rasouli N, Vickery EM, Sarnak M, Pittas AG; D2d Research Group; D2d Research Group collaborators. Effect of Vitamin D Supplementation on Kidney Function in Adults with Prediabetes: A Secondary Analysis of a Randomized Trial. Clin J Am Soc Nephrol. 2021 Aug;16(8):1201-1209. doi: 10.2215/CJN.00420121. | No safety data |
| 33 | Maghsoumi-Norouzabad L, Zare Javid A, Mansoori A, Dadfar M, Serajian A. The effects of Vitamin D3 supplementation on Spermatogram and endocrine factors in asthenozoospermia infertile men: a 15randomized, triple blind, placebo-controlled cli16nical trial. Reprod Biol Endocrinol. 2021 Jul 5;19(1):102. doi: 10.1186/s12958-021-00789-y. | Short duration |
| 34 | Rosser FJ, Han YY, Forno E, Bacharier LB, Phipatanakul W, Guilbert TW, Cabana MD, Ross K, Blatter J, Durrani S, Luther J, Wisniewski SR, Celedón JC. Effect of vitamin D supplementation on total and allergen-specific IgE in children with asthma and low vitamin D levels. J Allergy Clin Immunol. 2022 Jan;149(1):440-444.e2. doi: 10.1016/j.jaci.2021.05.037. Epub 2021 Jun 9. | Other publication of an included study |
| 35 | Fletcher J, Bedson E, Brown M, Hewison M, Swift A, Cooper SC. Protocol for an open-label feasibility study for a randomised controlled trial of vitamin D supplementation in Crohn's Disease patients with vitamin D deficiency: D-CODE Feasiblity study. Pilot Feasibility Stud. 2021 Mar 20;7(1):79. | Study protocol |
| 36 | Wanigatunga AA, Sternberg AL, Blackford AL, Cai Y, Mitchell CM, Roth DL, Miller ER 3rd, Szanton SL, Juraschek SP, Michos ED, Schrack JA, Appel LJ; STURDY Collaborative Research Group. The effects of vitamin D supplementation on types of falls. J Am Geriatr Soc. 2021 Oct;69(10):2851-2864. doi: 10.1111/jgs.17290. Epub 2021 Jun 12. | Other publication of an included study |
| 37 | Vinet A, Morrissey C, Perez-Martin A, Goncalves A, Raverdy C, Masson D, Gayrard S, Carrere M, Landrier JF, Amiot MJ. Effect of vitamin D supplementation on microvascular reactivity in obese adolescents: A randomized controlled trial. Nutr Metab Cardiovasc Dis. 2021 Jul 22;31(8):2474-2483. doi: 10.1016/j.numecd.2021.04.025. Epub 2021 May 10. | Short duration |
| 38 | Ben-Porat T, Weiss R, Khalaileh A, Abu Gazala M, Kaluti D, Mintz Y, Sherf-Dagan S, Yackobovitch-Gavan M, Rottenstreich A, Brodie R, Pikarsky AJ, Elazary R. The impact of preoperative vitamin administration on skeletal status following sleeve gastrectomy in young and middle-aged women: a randomized controlled trial. Int J Obes (Lond). 2021 Sep;45(9):1925-1936. doi: 10.1038/s41366-021-00845-y. Epub 2021 May 12. | Short duration |
| 39 | Miao J, Bachmann KN, Huang S, Su YR, Dusek J, Newton-Cheh C, Arora P, Wang TJ. Effects of Vitamin D Supplementation on Cardiovascular and Glycemic Biomarkers. J Am Heart Assoc. 2021 May 18;10(10):e017727. doi: 10.1161/JAHA.120.017727. Epub 2021 May 7. | Other publication of an included study |
| 40 | Sprague S, Bhandari M, Bzovsky S, Scott T, Thabane L, Heels-Ansdell D, O'Toole RV, Howe A, Gaski GE, Hill LC, Brown KM, Viskontas D, Zomar M, Della Rocca GJ, Slobogean GP. Fixation Using Alternative Implants for the Treatment of Hip Fractures: The feasibility of a multicenter 2 × 2 factorial randomized controlled trial evaluating surgical treatment and vitamin D supplementation in young femoral neck fracture patients OTA Int. 2020 May 21;3(2):e066. doi: 10.1097/OI9.0000000000000066. eCollection 2020 Jun. | No safety data |
| 41 | Chatterjee R, Fuss P, Vickery EM, LeBlanc ES, Sheehan PR, Lewis MR, Dolor RJ, Johnson KC, Kashyap SR, Nelson J, Pittas AG; D2d Research Group. Vitamin D Supplementation for Prevention of Cancer: The D2d Cancer Outcomes (D2dCA) Ancillary Study. J Clin Endocrinol Metab. 2021 Aug 18;106(9):2767-2778. doi: 10.1210/clinem/dgab153. | Other publication of an included study |
| 42 | Tan ML, Abrams SA, Osborn DA. Vitamin D supplementation for term breastfed infants to prevent vitamin D deficiency and improve bone health. Cochrane Database Syst Rev. 2020 Dec 11;12(12):CD013046. doi: 10.1002/14651858.CD013046.pub2. | Meta-analysis |
| 43 | Hu KL, Gan K, Wang R, Li W, Wu Q, Zheng B, Zou L, Zhang S, Liu Y, Wu Y, Chen R, Cao W, Yang S, Liu FT, Tian L, Zeng H, Xu H, Qiu S, Yang L, Chen X, Pan X, Wu X, Mol BW, Li R, Zhang D. Vitamin D supplementation prior to in vitro fertilisation in women with polycystic ovary syndrome: a protocol of a multicentre randomised, double-blind, placebo-controlled clinical trial. BMJ Open. 2020 Dec 8;10(12):e041409. doi: 10.1136/bmjopen-2020-041409. | Study protocol |
| 44 | Perić M, Maiter D, Cavalier E, Lasserre JF, Toma S. The Effects of 6-Month Vitamin D Supplementation during the Non-Surgical Treatment of Periodontitis in Vitamin-D-Deficient Patients: A Randomized Double-Blind Placebo-Controlled Study. Nutrients. 2020 Sep 25;12(10):2940. doi: 10.3390/nu12102940. | No 4000 IU daily |
| 45 | Wang L, Wen X, Lv S, Tian S, Jiang Y, Yang X. Effects of vitamin D supplementation on metabolic parameters of women with polycystic ovary syndrome: a meta-analysis of randomized controlled trials. Gynecol Endocrinol. 2021 May;37(5):446-455. doi: 10.1080/09513590.2020.1813272. Epub 2020 Sep 10. | Meta-analysis |
| 46 | van Vliet S, Fappi A, Reeds DN, Mittendorfer B. No independent or combined effects of vitamin D and conjugated linoleic acids on muscle protein synthesis in older adults: a randomized, double-blind, placebo-controlled clinical trial. Am J Clin Nutr. 2020 Nov 11;112(5):1382-1389. doi: 10.1093/ajcn/nqaa240. | Short duration |
| 47 | Burt LA, Billington EO, Rose MS, Kremer R, Hanley DA, Boyd SK. Adverse Effects of High-Dose Vitamin D Supplementation on Volumetric Bone Density Are Greater in Females than Males. J Bone Miner Res. 2020 Dec;35(12):2404-2414. doi: 10.1002/jbmr.4152. Epub 2020 Sep 16. | Other publication of an included study |
| 48 | Hashemian F, Sadegh S, Jahanshahi J, Seif Rabiei MA, Hashemian F. Effects of Vitamin D Supplementation on Recurrence of Nasal Polyposis after Endoscopic Sinus Surgery. Iran J Otorhinolaryngol. 2020 Jan;32(108):21-28. doi: 10.22038/ijorl.2019.37766.2241. | No safety data |
| 49 | Slobogean GP, Sprague S, Bzovsky S, Scott T, Thabane L, Heels-Ansdell D, O'Toole RV, Howe A, Gaski GE, Hill LC, Brown KM, Viskontas D, Zomar M, Della Rocca GJ, O'Hara NN, Bhandari M; FAITH-2 Investigators*. Fixation Using Alternative Implants for the Treatment of Hip Fractures (FAITH-2): The Clinical Outcomes of a Multicenter 2 × 2 Factorial Randomized Controlled Pilot Trial in Young Femoral Neck Fracture Patients. J Orthop Trauma. 2020 Oct;34(10):524-532. doi: 10.1097/BOT.0000000000001773. | No safety data |
| 50 | Rake C, Gilham C, Bukasa L, Ostler R, Newton M, Peto Wild J, Aigret B, Hill M, Gillie O, Nazareth I, Sasieni P, Martineau A, Peto J.High-dose oral vitamin D supplementation and mortality in people aged 65-84 years: the VIDAL cluster feasibility RCT of open versus double-blind individual randomisation. Health Technol Assess. 2020 Feb;24(10):1-54. | Bolus administration |
| 51 | Sudfeld CR, Mugusi F, Muhihi A, Aboud S, Nagu TJ, Ulenga N, Hong B, Wang M, Fawzi WW. Efficacy of vitamin D3 supplementation for the prevention of pulmonary tuberculosis and mortality in HIV: a randomised, double-blind, placebo-controlled trial. Lancet HIV. 2020 Jul;7(7):e463-e471. doi: 10.1016/S2352-3018(20)30108-9. | No 4000 IU daily |
| 52 | Meng H, Matthan NR, Angellotti E, Pittas AG, Lichtenstein AH. Exploring the effect of vitamin D3 supplementation on surrogate biomarkers of cholesterol absorption and endogenous synthesis in patients with type 2 diabetes-randomized controlled trial. Am J Clin Nutr. 2020 Sep 1;112(3):538-547. doi: 10.1093/ajcn/nqaa149. | Other publication of an included study |
| 53 | Billington EO, Burt LA, Plett R, Rose MS, Boyd SK, Hanley DA. Effect of high-dose vitamin D supplementation on peripheral arterial calcification: secondary analysis of a randomized controlled trial. Osteoporos Int. 2020 Nov;31(11):2141-2150. doi: 10.1007/s00198-020-05500-2. Epub 2020 Jun 15. | Other publication of an included study |
| 54 | Pérez-López FR, Pilz S, Chedraui P. Vitamin D supplementation during pregnancy: an overview. Curr Opin Obstet Gynecol. 2020 Oct;32(5):316-321. doi: 10.1097/GCO.0000000000000641. | Review |
| 55 | Soe HHK, Abas AB, Than NN, Ni H, Singh J, Said ARBM, Osunkwo I. Vitamin D supplementation for sickle cell disease. Cochrane Database Syst Rev. 2020 May 28;5(5):CD010858. doi: 10.1002/14651858.CD010858.pub3. | Review |
| 56 | Chen L, Dong Y, Bhagatwala J, Raed A, Huang Y, Zhu H. Vitamin D3 Supplementation Increases Long-Chain Ceramide Levels in Overweight/Obese African Americans: A Post-Hoc Analysis of a Randomized Controlled Trial. Nutrients. 2020 Apr 2;12(4):981. doi: 10.3390/nu12040981. | No low-dose vitamin D control |
| 57 | Burt LA, Gabel L, Billington EO, Hanley DA, Boyd SK. Postural Balance Effects Associated with 400, 4000 or 10,000 IU Vitamin D3 Daily for Three Years: A Secondary Analysis of a Randomized Clinical Trial. Nutrients. 2020 Feb 19;12(2):527. doi: 10.3390/nu12020527. | Other publication of an included study |
| 58 | Chen L, Wagner CL, Dong Y, Wang X, Shary JR, Huang Y, Hollis BW, Zhu H. Effects of Maternal Vitamin D3 Supplementation on Offspring Epigenetic Clock of Gestational Age at Birth: A Post-hoc Analysis of a Randomized Controlled Trial. Epigenetics. 2020 Aug;15(8):830-840. doi: 10.1080/15592294.2020.1734148. Epub 2020 Mar 1. | Other publication of an included study |
| 59 | Rashid A, Ganie MA, Wani IA, Bhat GA, Shaheen F, Wani IA, Shrivastava M, Shah ZA. Differential Impact of Insulin Sensitizers vs. Anti-Androgen on Serum Leptin Levels in Vitamin D Replete PCOS Women: A Six Month Open Labeled Randomized Study. Horm Metab Res. 2020 Feb;52(2):89-94. doi: 10.1055/a-1084-5441. Epub 2020 Feb 13. | No different vitamin D doses |
| 60 | Naderpoor N, Mousa A, Fernanda Gomez Arango L, Barrett HL, Dekker Nitert M, de Courten B. Effect of Vitamin D Supplementation on Faecal Microbiota: A Randomised Clinical Trial. Nutrients. 2019 Nov 27;11(12):2888. doi: 10.3390/nu11122888. | Short duration |
| 61 | Stoutjesdijk E, Schaafsma A, Kema IP, van der Molen J, Dijck-Brouwer DAJ, Muskiet FAJ. Influence of daily 10-85 μg vitamin D supplements during pregnancy and lactation on maternal vitamin D status and mature milk antirachitic activity. Br J Nutr. 2019 Feb;121(4):426-438. | Short duration |
| 62 | Shirvani A, Kalajian TA, Song A, Holick MF. Disassociation of Vitamin D's Calcemic Activity and Non-calcemic Genomic Activity and Individual Responsiveness: A Randomized Controlled Double-Blind Clinical Trial. Sci Rep. 2019 Nov 27;9(1):17685. doi: 10.1038/s41598-019-53864-1. | No low-dose vitamin D control |
| 63 | Sprague S, Bzovsky S, Connelly D, Thabane L, Adachi JD, Slobogean GP; Vita-Shock Investigators. Study protocol: design and rationale for an exploratory phase II randomized controlled trial to determine optimal vitamin D3 supplementation strategies for acute fracture healing. Pilot Feasibility Stud. 2019 Nov 22;5:135. doi: 10.1186/s40814-019-0524-4. eCollection 2019. | Study protocol |
| 64 | Amrein K, Parekh D, Westphal S, Preiser JC, Berghold A, Riedl R, Eller P, Schellongowski P, Thickett D, Meybohm P; VITDALIZE Collaboration Group. Effect of high-dose vitamin D3 on 28-day mortality in adult critically ill patients with severe vitamin D deficiency: a study protocol of a multicentre, placebo-controlled double-blind phase III RCT (the VITDALIZE study). BMJ Open. 2019 Nov 12;9(11):e031083. doi: 10.1136/bmjopen-2019-031083. | Study protocol |
| 65 | Chen L, Dong Y, Chen J, Huang Y, Zhu H. Epigenetics Predicts Serum 25-Hydroxyvitamin D Response to Vitamin D3 Supplementation in African Americans. Mol Nutr Food Res. 2020 Jan;64(1):e1900738. doi: 10.1002/mnfr.201900738. Epub 2019 Nov 8. | Short duration |
| 66 | Tedeschi SK, Aranow C, Kamen DL, LeBoff M, Diamond B, Costenbader KH. Effect of vitamin D on serum markers of bone turnover in SLE in a randomised controlled trial. Lupus Sci Med. 2019 Sep 17;6(1):e000352. doi: 10.1136/lupus-2019-000352. eCollection 2019. | Short duration |
| 67 | Gerveieeha Z, Siassi F, Qorbani M, Ziaeian F, Sotoudeh G. The effect of different amounts of vitamin D supplementation on serum calcidiol, anthropometric status, and body composition in overweight or obese nursing women: a study protocol for a randomized placebo-controlled clinical trial. Trials. 2019 Aug 30;20(1):542. doi: 10.1186/s13063-019-3622-y. | Short duration |
| 68 | Skrobot W, Liedtke E, Krasowska K, Dzik KP, Flis DJ, Samoraj-Dereszkiewicz A, Libionka W, Kortas J, Kloc W, Antosiewicz J, Kaczor JJ.Early Rehabilitation Program and Vitamin D Supplementation Improves Sensitivity of Balance and the Postural Control in Patients after Posterior Lumbar Interbody Fusion: A Randomized Trial. Nutrients. 2019 Sep 12;11(9):2202. | Short duration |
| 69 | Burt LA, Billington EO, Rose MS, Raymond DA, Hanley DA, Boyd SK. Effect of High-Dose Vitamin D Supplementation on Volumetric Bone Density and Bone Strength: A Randomized Clinical Trial. JAMA. 2019 Aug 27;322(8):736-745. doi: 10.1001/jama.2019.11889. | Other publication of an included study |
| 70 | Omidian M, Mahmoudi M, Javanbakht MH, Eshraghian MR, Abshirini M, Daneshzad E, Hasani H, Alvandi E, Djalali M. Effects of vitamin D supplementation on circulatory YKL-40 and MCP-1 biomarkers associated with vascular diabetic complications: A randomized, placebo-controlled, double-blind clinical trial. Diabetes Metab Syndr. 2019 Sep-Oct;13(5):2873-2877. doi: 10.1016/j.dsx.2019.07.047. Epub 2019 Jul 29. | Short duration |
| 71 | Omidian M, Mahmoudi M, Abshirini M, Eshraghian MR, Javanbakht MH, Zarei M, Hasani H, Djalali M. Effects of vitamin D supplementation on depressive symptoms in type 2 diabetes mellitus patients: Randomized placebo-controlled double-blind clinical trial. Diabetes Metab Syndr. 2019 Jul-Aug;13(4):2375-2380. doi: 10.1016/j.dsx.2019.06.011. Epub 2019 Jun 11. | Short duration |
| 72 | Javed Z, Papageorgiou M, Deshmukh H, Kilpatrick ES, Mann V, Corless L, Abouda G, Rigby AS, Atkin SL, Sathyapalan T.A Randomized, Controlled Trial of Vitamin D Supplementation on Cardiovascular Risk Factors, Hormones, and Liver Markers in Women with Polycystic Ovary Syndrome. Nutrients. 2019 Jan 17;11(1):188. | Short duration |
| 73 | FAITH-2 Investigators, Slobogean GP, Sprague S, Bzovsky S, Heels-Ansdell D, Thabane L, Scott T, Bhandari M. Fixation using alternative implants for the treatment of hip fractures (FAITH-2): design and rationale for a pilot multi-centre 2 × 2 factorial randomized controlled trial in young femoral neck fracture patients. Pilot Feasibility Stud. 2019 May 28;5:70. doi: 10.1186/s40814-019-0458-x. eCollection 2019. | Study protocol |
| 74 | Chatterjee R, Erban JK, Fuss P, Dolor R, LeBlanc E, Staten M, Sheehan P, Pittas A; D2d Research Group. Vitamin D supplementation for prevention of cancer: The D2d cancer outcomes (D2dCA) study. Contemp Clin Trials. 2019 Jun;81:62-70. doi: 10.1016/j.cct.2019.04.015. Epub 2019 Apr 29. | Study protocol |
| 75 | Rezagholizadeh F, Keshavarz SA, Djalali M, Rad EY, Alizadeh S, Javanbakht MH. Vitamin D3 supplementation improves serum SFRP5 and Wnt5a levels in patients with type 2 diabetes: A randomized, double-blind, placebo-controlled trial. Int J Vitam Nutr Res. 2018 Feb;88(1-2):73-79. doi: 10.1024/0300-9831/a000509. Epub 2019 Apr 10. | Short duration |
| 76 | Ebrahimpour-Koujan S, Sohrabpour AA, Foroughi F, Alvandi E, Esmaillzadeh A. Effects of vitamin D supplementation on liver fibrogenic factors in non-alcoholic fatty liver patients with steatohepatitis: study protocol for a randomized clinical trial. Trials. 2019 Mar 4;20(1):153. doi: 10.1186/s13063-019-3241-7. | Study protocol |
| 77 | Zittermann A, Ernst JB, Prokop S, Fuchs U, Gruszka A, Dreier J, Kuhn J, Knabbe C, Berthold HK, Gouni-Berthold I, Pilz S, Gummert JF, Paluszkiewicz L. Vitamin D supplementation of 4000 IU daily and cardiac function in patients with advanced heart failure: The EVITA trial. Int J Cardiol. 2019 Apr 1;280:117-123. doi: 10.1016/j.ijcard.2019.01.027. Epub 2019 Jan 9. | Other publication of an included study |
| 78 | Bassatne A, Chakhtoura M, Saad R, Fuleihan GE. Vitamin D supplementation in obesity and during weight loss: A review of randomized controlled trials. Metabolism. 2019 Mar;92:193-205. doi: 10.1016/j.metabol.2018.12.010. Epub 2019 Jan 4. | Review |
| 79 | Dzik K, Skrobot W, Flis DJ, Karnia M, Libionka W, Kloc W, Kaczor JJ. Vitamin D supplementation attenuates oxidative stress in paraspinal skeletal muscles in patients with low back pain. Eur J Appl Physiol. 2018 Jan;118(1):143-151. | Short duration |
| 80 | Rutjes AW, Denton DA, Di Nisio M, Chong LY, Abraham RP, Al-Assaf AS, Anderson JL, Malik MA, Vernooij RW, Martínez G, Tabet N, McCleery J. Vitamin and mineral supplementation for maintaining cognitive function in cognitively healthy people in mid and late life. Cochrane Database Syst Rev. 2018 Dec 17;12(12):CD011906. doi: 10.1002/14651858.CD011906.pub2. | Meta-analysis |
| 81 | Zittermann A, Ernst JB, Prokop S, Fuchs U, Dreier J, Kuhn J, Knabbe C, Börgermann J, Berthold HK, Pilz S, Gouni-Berthold I, Gummert JF. Daily Supplementation with 4000 IU Vitamin D3 for Three Years Does Not Modify Cardiovascular Risk Markers in Patients with Advanced Heart Failure: The Effect of Vitamin D on Mortality in Heart Failure Trial. Ann Nutr Metab. 2019;74(1):62-68. doi: 10.1159/000495662. Epub 2018 Dec 14. | Other publication of an included study |
| 82 | Enkhmaa D, Tanz L, Ganmaa D, Enkhtur S, Oyun-Erdene B, Stuart J, Chen G, Carr A, Seely EW, Fitzmaurice G, Buyandelger Y, Sarantsetseg B, Gantsetseg G, Rich-Edwards J. Randomized trial of three doses of vitamin D to reduce deficiency in pregnant Mongolian women. EBioMedicine. 2019 Jan;39:510-519. doi: 10.1016/j.ebiom.2018.11.060. Epub 2018 Dec 11. | No low-dose vitamin D control |
| 83 | Mesinovic J, Mousa A, Wilson K, Scragg R, Plebanski M, de Courten M, Scott D, Naderpoor N, de Courten B. Effect of 16-weeks vitamin D replacement on calcium-phosphate homeostasis in overweight and obese adults. J Steroid Biochem Mol Biol. 2019 Feb;186:169-175. doi: 10.1016/j.jsbmb.2018.10.011. Epub 2018 Oct 25. | Short duration |
| 84 | Angellotti E, D'Alessio D, Dawson-Hughes B, Chu Y, Nelson J, Hu P, Cohen RM, Pittas AG. Effect of vitamin D supplementation on cardiovascular risk in type 2 diabetes. Clin Nutr. 2019 Oct;38(5):2449-2453. doi: 10.1016/j.clnu.2018.10.003. Epub 2018 Oct 9. | Other publication of an included study |
| 85 | Mieszkowski J, Niespodziński B, Kochanowicz A, Gmiat A, Prusik K, Prusik K, Kortas J, Ziemann E, Antosiewicz J. The Effect of Nordic Walking Training Combined with Vitamin D Supplementation on Postural Control and Muscle Strength in Elderly People-A Randomized Controlled Trial. Int J Environ Res Public Health. 2018 Sep 7;15(9):1951. doi: 10.3390/ijerph15091951. | Short duration |
| 80 | Brady SRE, Naderpoor N, de Courten MPJ, Scragg R, Cicuttini F, Mousa A, de Courten B. Vitamin D supplementation may improve back pain disability in vitamin D deficient and overweight or obese adults. J Steroid Biochem Mol Biol. 2019 Jan;185:212-217. doi: 10.1016/j.jsbmb.2018.09.005. Epub 2018 Sep 7. | No 4000 IU vitamin D daily |
| 87 | Zhao JD, Jia JJ, Dong PS, Zhao D, Yang XM, Li DL, Zhang HF. Effect of vitamin D on ventricular remodelling in heart failure: a meta-analysis of randomised controlled trials. BMJ Open. 2018 Aug 30;8(8):e020545. doi: 10.1136/bmjopen-2017-020545. | Meta-analysis |
| 88 | Michos ED, Mitchell CM, Miller ER 3rd, Sternberg AL, Juraschek SP, Schrack JA, Szanton SL, Walston JD, Kalyani RR, Plante TB, Christenson RH, Shade D, Tonascia J, Roth DL, Appel LJ; STURDY Collaborative Research Group. Rationale and design of the Study To Understand Fall Reduction and Vitamin D in You (STURDY): A randomized clinical trial of Vitamin D supplement doses for the prevention of falls in older adults. Contemp Clin Trials. 2018 Oct;73:111-122. doi: 10.1016/j.cct.2018.08.004. Epub 2018 Aug 20. | Study protocol |
| 89 | Smith LM, Gallagher JC, Kaufmann M, Jones G. Effect of increasing doses of vitamin D on bone mineral density and serum N-terminal telopeptide in elderly women: a randomized controlled trial. J Intern Med. 2018 Dec;284(6):685-693. doi: 10.1111/joim.12825. Epub 2018 Sep 17. | Other publication of an included study |
| 90 | Zittermann A, Ernst JB, Prokop S, Fuchs U, Dreier J, Kuhn J, Knabbe C, Börgermann J, Berthold HK, Pilz S, Gouni-Berthold I, Gummert JF. Effects of Vitamin D Supplementation on Renin and Aldosterone Concentrations in Patients with Advanced Heart Failure: The EVITA Trial. Int J Endocrinol. 2018 Jul 3;2018:5015417. doi: 10.1155/2018/5015417. eCollection 2018. | Other publication of an included study |
| 91 | Brett NR, Gharibeh N, Weiler HA. Effect of Vitamin D Supplementation, Food Fortification, or Bolus Injection on Vitamin D Status in Children Aged 2-18 Years: A Meta-Analysis. Adv Nutr. 2018 Jul 1;9(4):454-464. doi: 10.1093/advances/nmy012. | Meta-analysis |
| 92 | Wright CS, Laing EM, Pollock NK, Hausman DB, Weaver CM, Martin BR, McCabe GP, Peacock M, Warden SJ, Hill Gallant KM, Lewis RD. Serum 25-Hydroxyvitamin D and Intact Parathyroid Hormone Influence Muscle Outcomes in Children and Adolescents. J Bone Miner Res. 2018 Nov;33(11):1940-1947. doi: 10.1002/jbmr.3550. Epub 2018 Aug 27. | Short duration |
| 93 | Bagheri M, Djazayery A, Qi L, Yekaninejad MS, Chamari M, Naderi M, Ebrahimi Z, Koletzko B, Uhl O, Farzadfar F. Effectiveness of vitamin D therapy in improving metabolomic biomarkers in obesity phenotypes: Two randomized clinical trials. Int J Obes (Lond). 2018 Oct;42(10):1782-1796. doi: 10.1038/s41366-018-0107-0. Epub 2018 Jun 11. | Short duration |
| 94 | De Niet S, Coffiner M, Da Silva S, Jandrain B, Souberbielle JC, Cavalier E. A Randomized Study to Compare a Monthly to a Daily Administration of Vitamin D₃ Supplementation. Nutrients. 2018 May 23;10(6):659. doi: 10.3390/nu10060659. | No 4000 IU vitamin D daily |
| 95 | Berlanga-Taylor AJ, Plant K, Dahl A, Lau E, Hill M, Sims D, Heger A, Emberson J, Armitage J, Clarke R, Knight JC. Genomic Response to Vitamin D Supplementation in the Setting of a Randomized, Placebo-Controlled Trial. EBioMedicine. 2018 May;31:133-142. doi: 10.1016/j.ebiom.2018.04.010. Epub 2018 Apr 10. | No safety data |
| 96 | Ali AM, Alobaid A, Malhis TN, Khattab AF. Effect of vitamin D3 supplementation in pregnancy on risk of pre-eclampsia - Randomized controlled trial. Clin Nutr. 2019 Apr;38(2):557-563. doi: 10.1016/j.clnu.2018.02.023. Epub 2018 Mar 2. | No safety data |
| 97 | Zittermann A, Ernst JB, Prokop S, Fuchs U, Dreier J, Kuhn J, Knabbe C, Berthold HK, Gouni-Berthold I, Gummert JF, Börgermann J, Pilz S. Vitamin D supplementation does not prevent the testosterone decline in males with advanced heart failure: the EVITA trial. Eur J Nutr. 2019 Mar;58(2):673-680. doi: 10.1007/s00394-018-1666-5. Epub 2018 Mar 15. | Other publication of an included study |
| 98 | Burt LA, Gaudet S, Kan M, Rose MS, Billington EO, Boyd SK, Hanley DA. Methods and procedures for: A randomized double-blind study investigating dose-dependent longitudinal effects of vitamin D supplementation on bone health. Contemp Clin Trials. 2018 Apr;67:68-73. doi: 10.1016/j.cct.2018.02.009. Epub 2018 Feb 20. | Study protocol |
| 99 | Zittermann A, Ernst JB, Prokop S, Fuchs U, Dreier J, Kuhn J, Berthold HK, Pilz S, Gouni-Berthold I, Gummert JF. Vitamin D supplementation and bone turnover in advanced heart failure: the EVITA trial. Osteoporos Int. 2018 Mar;29(3):579-586. doi: 10.1007/s00198-017-4312-9. Epub 2017 Dec 19. | Other publication of an included study |
| 100 | Raed A, Bhagatwala J, Zhu H, Pollock NK, Parikh SJ, Huang Y, Havens R, Kotak I, Guo DH, Dong Y. Dose responses of vitamin D3 supplementation on arterial stiffness in overweight African Americans with vitamin D deficiency: A placebo controlled randomized trial. PLoS One. 2017 Dec 7;12(12):e0188424. doi: 10.1371/journal.pone.0188424. eCollection 2017. | Bolus  administration |
| 101 | Jamilian M, Foroozanfard F, Rahmani E, Talebi M, Bahmani F, Asemi Z. Effect of Two Different Doses of Vitamin D Supplementation on Metabolic Profiles of Insulin-Resistant Patients with Polycystic Ovary Syndrome. Nutrients. 2017 Nov 24;9(12):1280. doi: 10.3390/nu9121280. | Short duration |
| 102 | Wei W, Shary JR, Garrett-Mayer E, Anderson B, Forestieri NE, Hollis BW, Wagner CL. Bone mineral density during pregnancy in women participating in a randomized controlled trial of vitamin D supplementation. Am J Clin Nutr. 2017 Dec;106(6):1422-1430. doi: 10.3945/ajcn.116.140459. Epub 2017 Oct 18. | Other publication of an included study |
| 103 | Riek AE, Oh J, Darwech I, Worthy V, Lin X, Ostlund RE Jr, Zhang RM, Bernal-Mizrachi C. Vitamin D3 supplementation decreases a unique circulating monocyte cholesterol pool in patients with type 2 diabetes. J Steroid Biochem Mol Biol. 2018 Mar;177:187-192. doi: 10.1016/j.jsbmb.2017.09.011. Epub 2017 Sep 21. | Short duration |
| 104 | Helde-Frankling M, Bergqvist J, Klasson C, Nordström M, Höijer J, Bergman P, Björkhem-Bergman L. Vitamin D supplementation to palliative cancer patients: protocol of a double-blind, randomised controlled trial 'Palliative-D'. BMJ Support Palliat Care. 2017 Dec;7(4):458-463. doi: 10.1136/bmjspcare-2017-001429. Epub 2017 Sep 13. | Study protocol |
| 105 | Ernst JB, Prokop S, Fuchs U, Dreier J, Kuhn J, Knabbe C, Berthold HK, Pilz S, Gouni-Berthold I, Gummert JF, Börgermann J, Zittermann A. Randomized supplementation of 4000 IU vitamin D3 daily vs placebo on the prevalence of anemia in advanced heart failure: the EVITA trial. Nutr J. 2017 Aug 23;16(1):49. doi: 10.1186/s12937-017-0270-5. | Other publication of an included study |
| 106 | Mousa A, Naderpoor N, de Courten MPJ, de Courten B. Vitamin D and symptoms of depression in overweight or obese adults: A cross-sectional study and randomized placebo-controlled trial. J Steroid Biochem Mol Biol. 2018 Mar;177:200-208. doi: 10.1016/j.jsbmb.2017.08.002. Epub 2017 Aug 10. | Short duration |
| 107 | Mousa A, Naderpoor N, de Courten MP, Teede H, Kellow N, Walker K, Scragg R, de Courten B. Vitamin D supplementation has no effect on insulin sensitivity or secretion in vitamin D-deficient, overweight or obese adults: a randomized placebo-controlled trial. Am J Clin Nutr. 2017 Jun;105(6):1372-1381. doi: 10.3945/ajcn.117.152736. Epub 2017 May 10. | Other publication of an included study |
| 108 | Smith LM, Gallagher JC, Suiter C. Medium doses of daily vitamin D decrease falls and higher doses of daily vitamin D3 increase falls: A randomized clinical trial. J Steroid Biochem Mol Biol. 2017 Oct;173:317-322. doi: 10.1016/j.jsbmb.2017.03.015. Epub 2017 Mar 18. | Other publication of an included study |
| 109 | Lotito A, Teramoto M, Cheung M, Becker K, Sukumar D. Serum Parathyroid Hormone Responses to Vitamin D Supplementation in Overweight/Obese Adults: A Systematic Review and Meta-Analysis of Randomized Clinical Trials. Nutrients. 2017 Mar 6;9(3):241. doi: 10.3390/nu9030241. | Meta-analysis |
| 110 | Muhammad J, Chan ES, Brown TT, Tebas P, McComsey GA, Melbourne K, Hardin R, Willig AL, Yin MT, Ribaudo H, Overton ET.Vitamin D Supplementation Does Not Affect Metabolic Changes Seen With ART Initiation. Open Forum Infect Dis. 2017 Dec 11;4(4):ofx210. doi: 10.1093/ofid/ofx210. eCollection 2017 Fall. | No safety data |
| 111 | Sudfeld CR, Mugusi F, Aboud S, Nagu TJ, Wang M, Fawzi WW. Efficacy of vitamin D3 supplementation in reducing incidence of pulmonary tuberculosis and mortality among HIV-infected Tanzanian adults initiating antiretroviral therapy: study protocol for a randomized controlled trial. Trials. 2017 Feb 10;18(1):66. doi: 10.1186/s13063-017-1819-5. | No 4000 IU vitamin D daily |
| 112 | Atkinson MA, Juraschek SP, Bertenthal MS, Detrick B, Furth SL, Miller ER 3rd. Pilot study of the effect of cholecalciferol supplementation on hepcidin in children with chronic kidney disease: Results of the D-fense Trial. Pediatr Nephrol. 2017 May;32(5):859-868. doi: 10.1007/s00467-016-3563-6. Epub 2016 Dec 24. | Short duration |
| 113 | Pop LC, Sukumar D, Schneider SH, Schlussel Y, Stahl T, Gordon C, Wang X, Papathomas TV, Shapses SA. Three doses of vitamin D, bone mineral density, and geometry in older women during modest weight control in a 1-year randomized controlled trial. Osteoporos Int. 2017 Jan;28(1):377-388. doi: 10.1007/s00198-016-3735-z. Epub 2016 Aug 17. | No low-dose vitamin D control |
| 114 | Cavalier E, Jandrain B, Coffiner M, Da Silva S, De Niet S, Vanderbist F, Souberbielle JC. A Randomised, Cross-Over Study to Estimate the Influence of Food on the 25-Hydroxyvitamin D₃ Serum Level after Vitamin D₃ Supplementation. Nutrients. 2016 May 20;8(5):309. doi: 10.3390/nu8050309. | No 4000 IU vitamin D daily |
| 115 | Alzaman NS, Dawson-Hughes B, Nelson J, D'Alessio D, Pittas AG. Vitamin D status of black and white Americans and changes in vitamin D metabolites after varied doses of vitamin D supplementation. Am J Clin Nutr. 2016 Jul;104(1):205-14. doi: 10.3945/ajcn.115.129478. Epub 2016 May 18. | Short duration |
| 116 | Chandler PD, Agboola F, Ng K, Scott JB, Drake BF, Bennett GG, Chan AT, Hollis BW, Emmons KM, Fuchs CS, Giovannucci EL. Reduction of Parathyroid Hormone with Vitamin D Supplementation in Blacks: A Randomized Controlled Trial. BMC Nutr. 2015;1:26. doi: 10.1186/s40795-015-0024-8. Epub 2015 Dec 17. | Short duration |
| 117 | Cassity EP, Redzic M, Teager CR, Thomas DT. The effect of body composition and BMI on 25(OH)D response in vitamin D-supplemented athletes. Eur J Sport Sci. 2016 Oct;16(7):773-9. doi: 10.1080/17461391.2015.1125952. Epub 2015 Dec 23. | No safety data |
| 118 | Konijeti GG, Arora P, Boylan MR, Song Y, Huang S, Harrell F, Newton-Cheh C, O'Neill D, Korzenik J, Wang TJ, Chan AT. Vitamin D Supplementation Modulates T Cell-Mediated Immunity in Humans: Results from a Randomized Control Trial. J Clin Endocrinol Metab. 2016 Feb;101(2):533-8. doi: 10.1210/jc.2015-3599. Epub 2015 Dec 14. | Other publication of an included study |
| 119 | Zaleski A, Panza G, Swales H, Arora P, Newton-Cheh C, Wang T, Thompson PD, Taylor B. High-Dose versus Low-Dose Vitamin D Supplementation and Arterial Stiffness among Individuals with Prehypertension and Vitamin D Deficiency. Dis Markers. 2015;2015:918968. doi: 10.1155/2015/918968. Epub 2015 Sep 16. | Other publication of an included study |
| 120 | Yousefi Rad E, Djalali M, Koohdani F, Saboor-Yaraghi AA, Eshraghian MR, Javanbakht MH, Saboori S, Zarei M, Hosseinzadeh-Attar MJ. The Effects of Vitamin D Supplementation on Glucose Control and Insulin Resistance in Patients with Diabetes Type 2: A Randomized Clinical Trial Study. Iran J Public Health. 2014 Dec;43(12):1651-6. | Short duration |
| 121 | Schall JI, Hediger ML, Zemel BS, Rutstein RM, Stallings VA. Comprehensive Safety Monitoring of 12-Month Daily 7000-IU Vitamin D3 Supplementation in Human Immunodeficiency Virus-Infected Children and Young Adults. JPEN J Parenter Enteral Nutr. 2016 Sep;40(7):1057-63. doi: 10.1177/0148607115593790. Epub 2015 Jul 9. | No 4000 IU vitamin D daily |
| 122 | Denlinger LC, King TS, Cardet JC, Craig T, Holguin F, Jackson DJ, Kraft M, Peters SP, Ross K, Sumino K, Boushey HA, Jarjour NN, Wechsler ME, Wenzel SE, Castro M, Avila PC; NHLBI AsthmaNet Investigators. Vitamin D Supplementation and the Risk of Colds in Patients with Asthma.  Am J Respir Crit Care Med. 2016 Mar 15;193(6):634-41. doi: 10.1164/rccm.201506-1169OC | No safety data |
| 123 | Schleck ML, Souberbielle JC, Jandrain B, Da Silva S, De Niet S, Vanderbist F, Scheen A, Cavalier E. A Randomized, Double-Blind, Parallel Study to Evaluate the Dose-Response of Three Different Vitamin D Treatment Schemes on the 25-Hydroxyvitamin D Serum Concentration in Patients with Vitamin D Deficiency. Nutrients. 2015 Jul 3;7(7):5413-22. doi: 10.3390/nu7075227. | Bolus administration |
| 124 | Chandler PD, Giovannucci EL, Scott JB, Bennett GG, Ng K, Chan AT, Hollis BW, Rifai N, Emmons KM, Fuchs CS, Drake BF. Effects of Vitamin D Supplementation on C-peptide and 25-hydroxyvitamin D Concentrations at 3 and 6 Months. Sci Rep. 2015 Jun 22;5:10411. doi: 10.1038/srep10411. | Short duration |
| 125 | Yeow TP, Lim SL, Hor CP, Khir AS, Wan Mohamud WN, Pacini G. Impact of Vitamin D Replacement on Markers of Glucose Metabolism and Cardio-Metabolic Risk in Women with Former Gestational Diabetes--A Double-Blind, Randomized Controlled Trial. PLoS One. 2015 Jun 9;10(6):e0129017. doi: 10.1371/journal.pone.0129017. eCollection 2015. | No safety data |
| 126 | Dougherty KA, Bertolaso C, Schall JI, Smith-Whitley K, Stallings VA. Safety and Efficacy of High-dose Daily Vitamin D3 Supplementation in Children and Young Adults With Sickle Cell Disease. J Pediatr Hematol Oncol. 2015 Jul;37(5):e308-15. doi: 10.1097/MPH.0000000000000355. | Short duration |
| 127 | Rodda CP, Benson JE, Vincent AJ, Whitehead CL, Polykov A, Vollenhoven B. Maternal vitamin D supplementation during pregnancy prevents vitamin D deficiency in the newborn: an open-label randomized controlled trial. Clin Endocrinol (Oxf). 2015 Sep;83(3):363-8. doi: 10.1111/cen.12762. Epub 2015 Apr 8. | No safety data |
| 128 | Steenhoff AP, Schall JI, Samuel J, Seme B, Marape M, Ratshaa B, Goercke I, Tolle M, Nnyepi MS, Mazhani L, Zemel BS, Rutstein RM, Stallings VA. Vitamin D₃supplementation in Batswana children and adults with HIV: a pilot double blind randomized controlled trial. PLoS One. 2015 Feb 23;10(2):e0117123. doi: 10.1371/journal.pone.0117123. eCollection 2015. | Short duration |
| 129 | Foresta C, Calogero AE, Lombardo F, Lenzi A, Ferlin A. Late-onset hypogonadism: beyond testosterone. Asian J Androl. 2015 Mar-Apr;17(2):236-8. doi: 10.4103/1008-682X.135985. | Short duration |
| 130 | Tomlinson PB, Joseph C, Angioi M. Effects of vitamin D supplementation on upper and lower body muscle strength levels in healthy individuals. A systematic review with meta-analysis. J Sci Med Sport. 2015 Sep;18(5):575-80. doi: 10.1016/j.jsams.2014.07.022. Epub 2014 Aug 11. | Meta-analysis |
| 131 | Dalbeni A, Scaturro G, Degan M, Minuz P, Delva P. Effects of six months of vitamin D supplementation in patients with heart failure: a randomized double-blind controlled trial. Nutr Metab Cardiovasc Dis. 2014 Aug;24(8):861-8. doi: 10.1016/j.numecd.2014.02.015. Epub 2014 Mar 5. | Bolus administration |
| 132 | Chandler PD, Scott JB, Drake BF, Ng K, Forman JP, Chan AT, Bennett GG, Hollis BW, Giovannucci EL, Emmons KM, Fuchs CS. Risk of hypercalcemia in blacks taking hydrochlorothiazide and vitamin D. Am J Med. 2014 Aug;127(8):772-8. doi: 10.1016/j.amjmed.2014.02.044. Epub 2014 Mar 20. | Short duration |
| 133 | Hossain N, Kanani FH, Ramzan S, Kausar R, Ayaz S, Khanani R, Pal L. Obstetric and neonatal outcomes of maternal vitamin D supplementation: results of an open-label, randomized controlled trial of antenatal vitamin D supplementation in Pakistani women. J Clin Endocrinol Metab. 2014 Jul;99(7):2448-55. doi: 10.1210/jc.2013-3491. Epub 2014 Mar 19. | Short duration |
| 134 | Aloia JF, Dhaliwal R, Shieh A, Mikhail M, Fazzari M, Ragolia L, Abrams SA. Vitamin D supplementation increases calcium absorption without a threshold effect. Am J Clin Nutr. 2014 Mar;99(3):624-31. doi: 10.3945/ajcn.113.067199. Epub 2013 Dec 11. | No low-dose vitamin D control |
| 135 | Ng K, Scott JB, Drake BF, Chan AT, Hollis BW, Chandler PD, Bennett GG, Giovannucci EL, Gonzalez-Suarez E, Meyerhardt JA, Emmons KM, Fuchs CS. Dose response to vitamin D supplementation in African Americans: results of a 4-arm, randomized, placebo-controlled trial. Am J Clin Nutr. 2014 Mar;99(3):587-98. doi: 10.3945/ajcn.113.067777. Epub 2013 Dec 24. | Short duration |
| 136 | Nieman DC, Gillitt ND, Shanely RA, Dew D, Meaney MP, Luo B. Vitamin D2 supplementation amplifies eccentric exercise-induced muscle damage in NASCAR pit crew athletes. Nutrients. 2013 Dec 20;6(1):63-75. | Short duration |
| 137 | Ceglia L, Niramitmahapanya S, da Silva Morais M, Rivas DA, Harris SS, Bischoff-Ferrari H, Fielding RA, Dawson-Hughes B. A randomized study on the effect of vitamin D₃ supplementation on skeletal muscle morphology and vitamin D receptor concentration in older women. J Clin Endocrinol Metab. 2013 Dec;98(12):E1927-35. doi: 10.1210/jc.2013-2820. Epub 2013 Oct 9. | Short duration |
| 138 | Lewis RD, Laing EM, Hill Gallant KM, Hall DB, McCabe GP, Hausman DB, Martin BR, Warden SJ, Peacock M, Weaver CM. A randomized trial of vitamin D₃ supplementation in children: dose-response effects on vitamin D metabolites and calcium absorption. J Clin Endocrinol Metab. 2013 Dec;98(12):4816-25. doi: 10.1210/jc.2013-2728. Epub 2013 Oct 3. | Short duration |
| 112 | Hata TR, Audish D, Kotol P, Coda A, Kabigting F, Miller J, Alexandrescu D, Boguniewicz M, Taylor P, Aertker L, Kesler K, Hanifin JM, Leung DY, Gallo RL. A randomized controlled double-blind investigation of the effects of vitamin D dietary supplementation in subjects with atopic dermatitis. J Eur Acad Dermatol Venereol. 2014 Jun;28(6):781-9. doi: 10.1111/jdv.12176. Epub 2013 May 3. | Short duration |
| 139 | Mehrotra A, Calvo MS, Beelman RB, Levy E, Siuty J, Kalaras MD, Uribarri J. Bioavailability of vitamin D2 from enriched mushrooms in prediabetic adults: a randomized controlled trial. Eur J Clin Nutr. 2014 Oct;68(10):1154-60. doi: 10.1038/ejcn.2014.157. | Short duration |
| 140 | Dawodu A, Saadi HF, Bekdache G, Javed Y, Altaye M, Hollis BW. Randomized controlled trial (RCT) of vitamin D supplementation in pregnancy in a population with endemic vitamin D deficiency. J Clin Endocrinol Metab. 2013 Jun;98(6):2337-46. doi: 10.1210/jc.2013-1154. Epub 2013 Apr 4. | No safety data |
| 141 | Björkhem-Bergman L, Nylén H, Norlin AC, Lindh JD, Ekström L, Eliasson E, Bergman P, Diczfalusy U. Serum levels of 25-hydroxyvitamin D and the CYP3A biomarker 4β-hydroxycholesterol in a high-dose vitamin D supplementation study. Drug Metab Dispos. 2013 Apr;41(4):704-8. doi: 10.1124/dmd.113.051136. Epub 2013 Feb 5. | Other publication of an included study |
| 142 | Gallagher JC, Peacock M, Yalamanchili V, Smith LM. Effects of vitamin D supplementation in older African American women. J Clin Endocrinol Metab. 2013 Mar;98(3):1137-46. doi: 10.1210/jc.2012-3106. Epub 2013 Feb 5. | Small number of participants |
| 143 | Wagner CL, McNeil R, Hamilton SA, Winkler J, Rodriguez Cook C, Warner G, Bivens B, Davis DJ, Smith PG, Murphy M, Shary JR, Hollis BW. A randomized trial of vitamin D supplementation in 2 community health center networks in South Carolina. Am J Obstet Gynecol. 2013 Feb;208(2):137.e1-13. doi: 10.1016/j.ajog.2012.10.888. Epub 2012 Nov 3. | No low-dose vitamin D control |
| 144 | Groleau V, Herold RA, Schall JI, Wagner JL, Dougherty KA, Zemel BS, Rutstein RM, Stallings VA. Blood lead concentration is not altered by high-dose vitamin D supplementation in children and young adults with HIV. J Pediatr Gastroenterol Nutr. 2013 Mar;56(3):316-9. doi: 10.1097/MPG.0b013e3182758c4a. | No low-dose vitamin D control |
| 145 | Carrillo AE, Flynn MG, Pinkston C, Markofski MM, Jiang Y, Donkin SS, Teegarden D. Impact of vitamin D supplementation during a resistance training intervention on body composition, muscle function, and glucose tolerance in overweight and obese adults. Clin Nutr. 2013 Jun;32(3):375-81. doi: 10.1016/j.clnu.2012.08.014. Epub 2012 Aug 31. | Short duration |
| 146 | Harris SS, Pittas AG, Palermo NJ. A randomized, placebo-controlled trial of vitamin D supplementation to improve glycaemia in overweight and obese African Americans. Diabetes Obes Metab. 2012 Sep;14(9):789-94. doi: 10.1111/j.1463-1326.2012.01605.x. Epub 2012 May 8. | Short duration |
| 147 | Hollis BW, Wagner CL. Vitamin D requirements and supplementation during pregnancy. Curr Opin Endocrinol Diabetes Obes. 2011 Dec;18(6):371-5. doi: 10.1097/MED.0b013e32834b0040. | Review |
| 148 | Roth DE. Vitamin D supplementation during pregnancy: safety considerations in the design and interpretation of clinical trials. J Perinatol. 2011 Jul;31(7):449-59. doi: 10.1038/jp.2010.203. Epub 2011 Jan 20. | No RCT |
| 149 | von Hurst PR, Stonehouse W, Kruger MC, Coad J. Vitamin D supplementation suppresses age-induced bone turnover in older women who are vitamin D deficient. J Steroid Biochem Mol Biol. 2010 Jul;121(1-2):293-6. doi: 10.1016/j.jsbmb.2010.03.054. Epub 2010 Mar 19. | No safety data |
| 150 | von Hurst PR, Stonehouse W, Coad J. Vitamin D supplementation reduces insulin resistance in South Asian women living in New Zealand who are insulin resistant and vitamin D deficient - a randomised, placebo-controlled trial. Br J Nutr. 2010 Feb;103(4):549-55. doi: 10.1017/S0007114509992017. Epub 2009 Sep 28. | No safety data |
| 151 | Wagner D, Sidhom G, Whiting SJ, Rousseau D, Vieth R. The bioavailability of vitamin D from fortified cheeses and supplements is equivalent in adults. J Nutr. 2008 Jul;138(7):1365-71. doi: 10.1093/jn/138.7.1365. | No 4000 IU vitamin D dose |
| 152 | Aloia JF, Patel M, Dimaano R, Li-Ng M, Talwar SA, Mikhail M, Pollack S, Yeh JK. Vitamin D intake to attain a desired serum 25-hydroxyvitamin D concentration. Am J Clin Nutr. 2008 Jun;87(6):1952-8. | Dosing regimen does not meet inclusion criteria |
| 153 | Talwar SA, Aloia JF, Pollack S, Yeh JK. Dose response to vitamin D supplementation among postmenopausal African American women. Am J Clin Nutr. 2007 Dec;86(6):1657-62. doi: 10.1093/ajcn/86.5.1657. | No 4000 IU vitamin D dose |
| 154 | Hollis BW, Wagner CL. Vitamin D requirements during lactation: high-dose maternal supplementation as therapy to prevent hypovitaminosis D for both the mother and the nursing infant. Am J Clin Nutr. 2004 Dec;80(6 Suppl):1752S-8S. doi: 10.1093/ajcn/80.6.1752S. | Short duration |
| 155 | Basile LA, Taylor SN, Wagner CL, Horst RL, Hollis BW. The effect of high-dose vitamin D supplementation on serum vitamin D levels and milk calcium concentration in lactating women and their infants. Breastfeed Med. 2006 Spring;1(1):27-35. doi: 10.1089/bfm.2006.1.27. | Short duration |
| 156 | Vieth R, Kimball S, Hu A, Walfish PG. Randomized comparison of the effects of the vitamin D3 adequate intake versus 100 mcg (4000 IU) per day on biochemical responses and the wellbeing of patients. Nutr J. 2004 Jul 19;3:8. doi: 10.1186/1475-2891-3-8 | No low-dose control |
| 157 | Hollis BW, Wagner CL. Vitamin D requirements during lactation: high-dose maternal supplementation as therapy to prevent hypovitaminosis D for both the mother and the nursing infant. Clinical Trial Am J Clin Nutr . 2004 Dec;80(6 Suppl):1752S-8S. | Short duration |

**Supplemental Figure 1:** Subgroup analyses of vitamin D vs. control on the relative risk of hypercalcemia


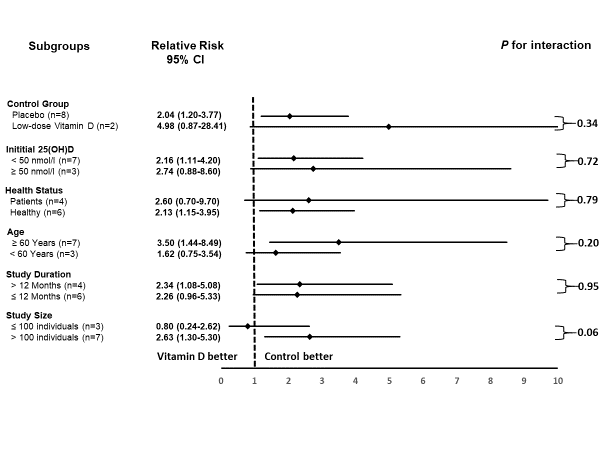


Figure legend: Diamonds represent the mean, and error bars indicate 95% confidence intervals of the relative risk in subgroups of randomized clinical trials. Numbers indicate number of trials in each subgroup, and P values refer to differences between subgroups. Abbreviations: CI, confidence interval; 25(OH)D, 25-hydroxyvitamin D

**Supplemental Figure 2:** Funnel plot of included studies for the assessment of the risk of hypercalcemia


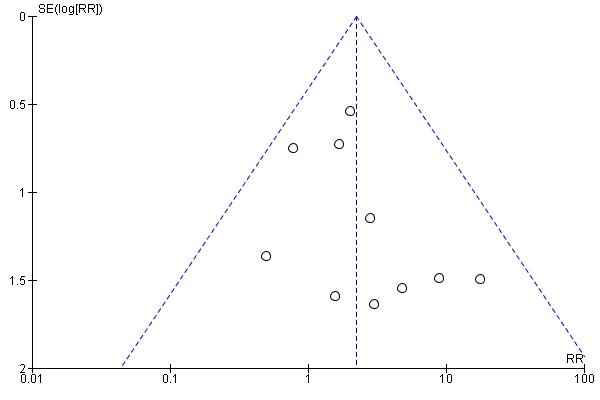


Figure legend: RR indicates the relative risk of vitamin D vs. control in individual trials; each circle displays the result of an individual study; a value < 1 notifies a decreased risk and a value > 1 notifies an increased risk by vitamin D supplementation; SE (log [RR) denotes the standard error of the log-transformed relative risk. Risk of publication bias cannot be ruled out if circles are lying outside the dotted lines.

**Supplemental Figure 3:** Funnel plot of included studies for the assessment of the risk of hypercalciuria


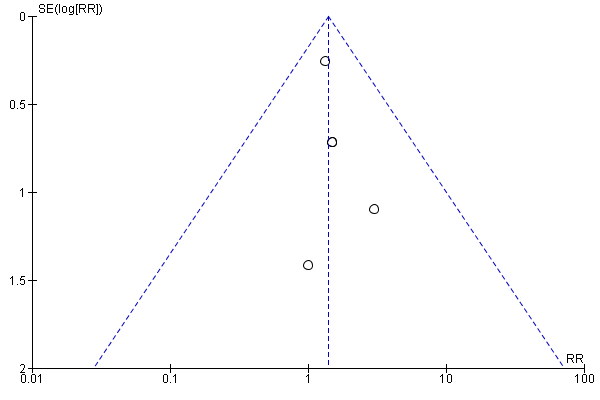


Figure legend: RR indicates the relative risk of vitamin D vs. control in individual trials; each circle displays the result of an individual study; a value < 1 notifies a decreased risk and a value > 1 notifies an increased risk by vitamin D supplementation; SE (log [RR) denotes the standard error of the log-transformed relative risk. Risk of publication bias cannot be ruled out if circles are lying outside the dotted lines.

**Supplemental Figure 4:** Funnel plot of included studies for the assessment of the risk of kidney stones


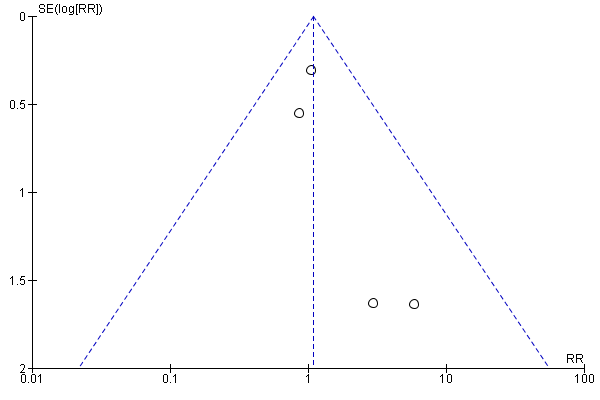


Figure legend: RR indicates the relative risk of vitamin D vs. control in individual trials; each circle displays the result of an individual study; a value < 1 notifies a decreased risk and a value > 1 notifies an increased risk by vitamin D supplementation; SE (log [RR) denotes the standard error of the log-transformed relative risk. Risk of publication bias cannot be ruled out if circles are lying outside the dotted lines.

**Supplemental Figure 5:** Funnel plot of included studies for the assessment of the risk of falls


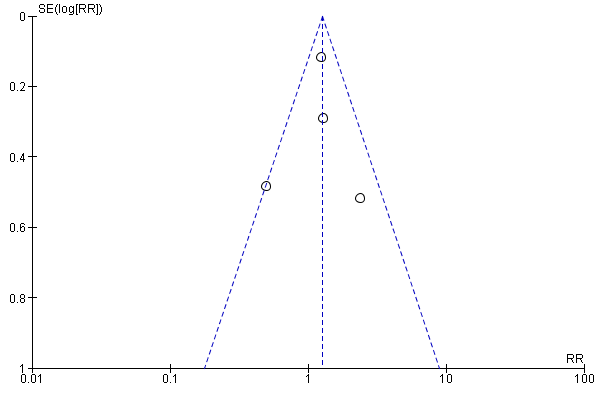


Figure legend: RR indicates the relative risk of vitamin D vs. control in individual trials; each circle displays the result of an individual study; a value < 1 notifies a decreased risk and a value > 1 notifies an increased risk by vitamin D supplementation; SE (log [RR) denotes the standard error of the log-transformed relative risk. Risk of publication bias cannot be ruled out if circles are lying outside the dotted lines.

**Supplemental Figure 6:** Funnel plot of included studies for the assessment of the risk of hospitalization


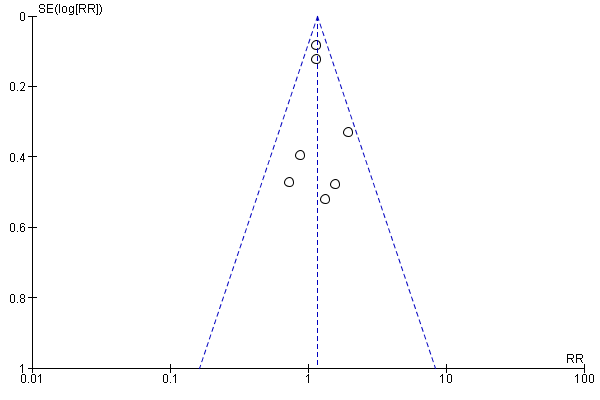


Figure legend: RR indicates the relative risk of vitamin D vs. control in individual trials; each circle displays the result of an individual study; a value < 1 notifies a decreased risk and a value > 1 notifies an increased risk by vitamin D supplementation; SE (log [RR) denotes the standard error of the log-transformed relative risk. Risk of publication bias cannot be ruled out if circles are lying outside the dotted lines.

**Supplemental Figure 7:** Funnel plot of included studies for the assessment of the risk of mortality


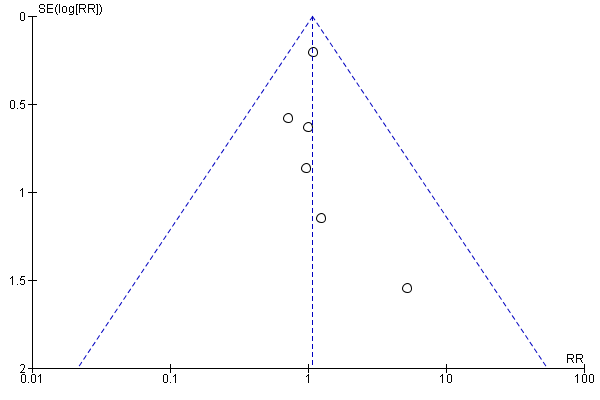


Figure legend: RR indicates the relative risk of vitamin D vs. control in individual trials; each circle displays the result of an individual study; a value < 1 notifies a decreased risk and a value > 1 notifies an increased risk by vitamin D supplementation; SE (log [RR) denotes the standard error of the log-transformed relative risk. Risk of publication bias cannot be ruled out if circles are lying outside the dotted lines.

**Supplemental Figure 8:** Methodological quality graph: authors´ judgments about each methodological quality item regarding adverse events presented as percentages for included studies


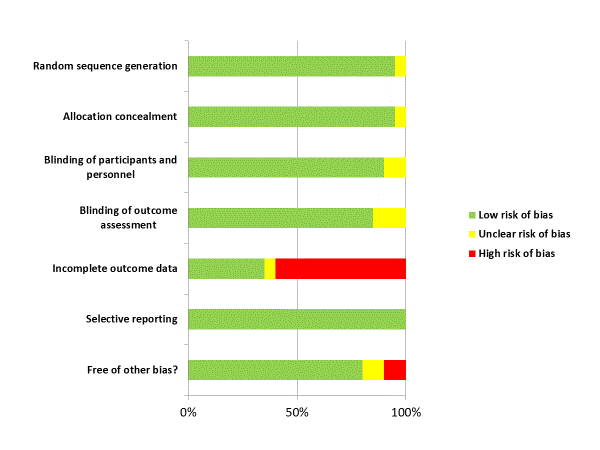

Supplement: Supplementary file 1 — Supplementary file1 (DOCX 127 KB) [file 394_2023_3124_MOESM1_ESM.docx]
